# Stimuli-responsive hydrogels based on cascade reactions: a novel strategy to promote the efficient repair of diabetic wounds

**DOI:** 10.7150/thno.126282

**Published:** 2026-01-01

**Authors:** Jialin Jia, Xiaosu Wang, Tao Zhang, Qingxia Sun, Shude Yang, Wenna Wu

**Affiliations:** 1 School of Chemistry and Chemical Engineering, Yantai University, Yantai 264005, Shandong Province, PR China.; 2 Center of Plastic and Cosmetic Surgery, School and Hospital of Stomatology, China Medical University, Liaoning Provincial Key Laboratory of Oral Diseases, Shenyang 110001, Liaoning Province, PR China.

**Keywords:** cascade reaction, hydrogel, responsiveness, diabetic wound

## Abstract

The impaired healing of diabetic wounds is caused by complex multifactorial pathologies and conventional therapeutic approaches often show limited efficacy. In recent years, stimulus-responsive hydrogels based on cascade reactions have become a promising approach in the management of diabetic wounds. These hydrogels are designed to react to particular characteristics of the wound microenvironment, such as glucose concentration, pH, reactive oxygen species (ROS) and enzyme activity, allowing spatiotemporally controlled drug release and synergistic multi-target control. This review focuses on the recent development in understanding of the pathophysiology of diabetic wounds, immune microenvironment modulation, and the development of stimuli-responsive cascade hydrogels, as well as the challenges. By integrating responsive moieties, these hydrogels dynamically control the polarization of immune cells and scavenging of ROS. Furthermore, cascade systems, from single-step to multistep design, enable precise spatiotemporal activation and coordinate antibacterial, antioxidant and pro-regenerative effects. Additionally, emerging technologies such as AI-assisted modeling, biosensing-guided feedback, and organ-on-a-chip platforms have great potential to improve the rational design and predictive validation of cascade hydrogel systems, paving the way for intelligent and personalized diabetic wound therapies.

## 1. Introduction

Diabetes mellitus (DM) is a chronic metabolic disorder that is spreading across the globe at an unprecedented rate. According to the international diabetes federation, the number of people suffering from diabetes is rapidly rising and is expected to reach almost 1.31 billion by 2050 1. Beyond greatly affecting the quality of life of patients, diabetes leads to many complications, such as cardiovascular and cerebrovascular diseases, nephropathy, retinopathy and neuropathy, that impose a significant burden on the healthcare systems worldwide 2,3. Among these complications, the wound healing of diabetics is a particularly formidable challenge, with a long healing process, risk of infection and a high rate of recurrence 4. In severe cases, wounds may become chronic and non-healing and may lead to amputation.

The diabetic wound healing process is hindered by several interrelated factors including persistent inflammation, hyperglycemia, excessive reactive oxygen species (ROS), bacterial infections, hypoxia and other complex microenvironmental disturbances 5. These obstacles hinder the normal repair cascade, thus highlighting the urgent need for efficacious therapeutic strategies against diabetic wounds. Wound dressings play an integral part in treatment and create an essential barrier and healing 6. However, conventional dressings, such as gauze and foam, mostly maintain moisture and prevent bacterial contamination but are limited in their functional capabilities. They do not adequately address the multifaceted and dynamic microenvironmental alterations that are characteristic of diabetic wounds, and therefore do not adequately address the diverse and evolving needs of this patient population 5.

In the last few years, hydrogels have become a promising new class of wound dressings with a great potential in treating diabetic wounds. Hydrogels are composed of hydrophilic polymer networks that can absorb and hold large volumes of water, which in turn provides a moist environment that can enhance healing while minimizing bacterial colonization. Compared to traditional dressings, hydrogels more closely replicate the structure of the extracellular matrix (ECM) providing better cell adhesion and migration, which increases wound repair. Moreover, the high tunability of the hydrogels enables customization depending on the specific circumstances of the wound, which enables tailored treatment strategies to address diverse treatment requirements 7-9. Stimuli-responsive hydrogels have attracted broad attention because of their intelligent, adaptive characteristics 10. These hydrogels can react to external stimuli, such as pH, temperature, enzyme activity, or biomolecular signals, by changing their physical and chemical properties to adapt to the changing wound microenvironment. For example, pH-responsive hydrogels can respond to the acidic environment common to the inflammatory phase and release anti-inflammatory agents to reduce inflammation. Similarly, temperature-responsive hydrogels change their hardness or shape depending on the body temperature and hence can better conform to the wound contour 11-13. These smart, stimuli-responsive behaviors facilitate active and enhanced hydrogel involvement in the wound healing process and provide novel strategies to handle diabetic wounds.

Cascade reactions are frequently found in biology as a series of consecutive chemical reactions, in which the product of one reaction is a catalyst for the next 14-15. In tissue repair this concept has been exploited to design smart materials that can mimic these complex biological processes 16-18. By integrating the responsive units into the hydrogel networks, the cascade reactions can be induced to experience a sequence of orderly transformations in response to specific stimuli, thus allowing the wound microenvironment to be precisely regulated.

**Figure 1:**
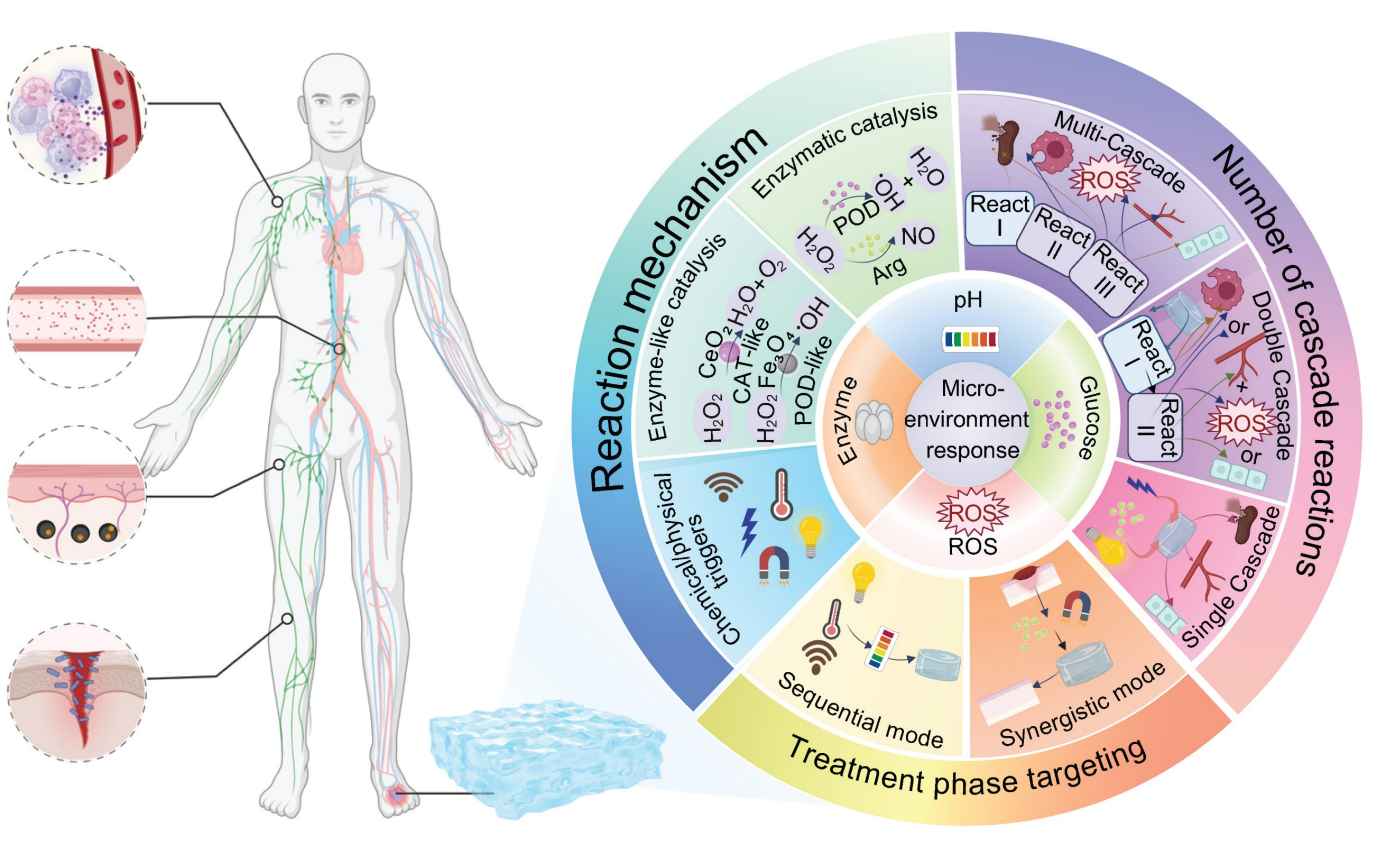
Schematic representation of the pathological characteristics of diabetic wound healing and the types and classification criteria of the cascade reactions contained in microenvironment-responsive hydrogels. Created in BioRender. Jia, J. (2025) https://BioRender.com/b8q7n94.

This review focuses on giving a comprehensive summary of diabetic wound pathology and wound therapeutic strategies, especially focusing on the recent progress of stimulus-responsive hydrogels based on cascade reactions. First, the pathophysiological process in diabetic wounds is explained and divided into four different stages: hemostasis, inflammation, proliferation, and remodeling. Emphasis is placed on the important roles of macrophages and neutrophils in modulating the immune microenvironment during each stage as well as the relevant cell signaling pathways. Next, combining these cellular and molecular knowledge, several types of microenvironment-responsive hydrogels, such as glucose-, pH-, enzyme-, and ROS-responsive systems are discussed for their ability to enhance the local microenvironment by facilitating neovascularization, tissue repair, and immune regulation (**Figure 1**). Based on the source of stimulation, various cascade hydrogel systems have been developed, with most of the current models based on the principle of enzymatic cascade. Increasingly, dual or multi-step modules are used to improve the spatiotemporal precision. These approaches have a lot of benefits including enhanced catalytic efficiency and stability, especially in nanozyme mimicking systems, as well as enabling synergistic cascades to promote diabetic wound therapy towards complete and dynamic regulation. Finally, the review emphasizes outstanding challenges such as the development of intelligent materials that are able to dynamically adapt to different healing stages, the coordination of multiple simultaneous response signals, and the achievement of an optimal balance between biosafety and therapeutic functionality. Future research directions may include organ-on-a-chip (OoC) platforms to simulate wound microenvironments, minimizing off-target effects in biomimetic systems, and creating multiscale frameworks for evaluating biocompatibility. A more integrated approach that combines mechanistic understanding, materials innovation and clinical validation is critical for the advancement of precision and personalized management of diabetic wounds.

## 2. Pathophysiology of Diabetic Wound Healing

### 2.1 Characteristics of diabetic wounds and their impact on each stage of wound healing

Diabetic wounds are defined by their chronicity and resistance to healing 1. The pathophysiology behind this is chronic inflammation, poor angiogenesis and susceptibility to bacterial infection. The hyperglycemic microenvironment enhances excessive accumulation of ROS, which induces sustained expression of pro-inflammatory factors, thus continuing a vicious cycle of inflammation 4, 6. Additionally, hyperglycemia causes damage to the vascular endothelial cells, resulting in impaired neovascularization and chronic local hypoxia in the wound 4. Elevated glucose levels also support the growth of pathogenic bacteria, which greatly raises the risk of infection 10. Moreover, peripheral neuropathy has been shown to interfere with the tissue repair process, further complicating wound healing in diabetic patients 19, 20.

Abnormally prolonged inflammatory phase: In normal wound healing, the inflammatory phase is normally short. However, in diabetic wounds this response is unable to resolve on time, as a result of macrophage dysfunction 21, 22. Notably, the expression of netrin-1 protein is significantly reduced in diabetic wounds, which affects the polarization of macrophages into the anti-inflammatory M2 phenotype. Concurrently, pro-inflammatory cytokines like TNF-α and IL-6 are continuously secreted and create a pathological inflammatory microenvironment 21, 23.

Impaired proliferative phase function: Persistent hyperglycemia has been demonstrated to suppress fibroblast activity, leading to decreased synthesis and disordered structure of the ECM 24. Oxidative stress, which is associated with excessive production of ROS, further impair keratinocyte migration and slows epithelialization 25. The vascular endothelial growth factor (VEGF) signaling pathway is inhibited, worsening the hypoxia in the tissues with the lack of blood vessel formation 26. Moreover, impaired expression of pro-angiogenic factors such as Cu^2+^ and netrin-1 affects the proliferation of endothelial cells in diabetic wounds 27, 28.

Structural defects during remodeling: An imbalance in collagen cross-linking causes reduced scar strength 25. The prolonged action of matrix metalloproteinases (MMPs) leads to the breakdown of newly formed tissue in excess, making wounds prone to recurrence or deterioration 29.

The diabetic wound microenvironment is defined by a complex interplay of pathological factors. Hypoxia and oxidative stress are reciprocally related: hypoxia leads to increased ROS generation, which further accumulates and suppresses the activity of hypoxia-inducible factor-1 alpha (HIF-1α), leading to a vicious cycle 30, 31. Likewise, infection and inflammation act in a synergistic manner to increase tissue damage with bacterial biofilms enhancing the inflammatory response whereas proteases released by inflammatory cells degrade the antimicrobial barrier 5, 32.

Conventional therapeutic modalities often have difficulty modulating these multifaceted factors simultaneously 33. Recent advances have revealed novel strategies that target macrophage phenotype switching, for example, topical administration of exosomes (Exos) has been demonstrated to promote macrophage polarization towards the M2 phenotype 34. In addition, the dual control of oxidative stress and angiogenesis by nano-enzymatic materials has proven to be promising 35. Metal-organic framework (MOF)-based catalysts have shown the potential of scavenging ROS and facilitating vascular regeneration 36. Furthermore, smart-responsive dressings, such as glucose-sensitive hydrogels allow dynamic drug release based on microenvironment changes in the wound 6, 10.

Given the multifactorial and interactive nature of diabetic wounds, pathological deviations occur in the various phases of the inflammatory, proliferative and remodeling phases of healing. Future studies should focus on the development of multi-target synergistic intervention strategies designed to restore and restructure the wound microenvironment in order to promote effective healing.

### 2.2 The important regulatory role of the immune microenvironment (macrophages, neutrophils) in each stage of diabetic wound repair

The skin immune microenvironment is a complex network of cells including neutrophils, macrophages, T cells, B cells and natural killer (NK) cells that interact closely with keratinocytes and fibroblasts to maintain tissue homeostasis and defend against pathogens 37-39. These immune cells communicate mainly via cytokines, orchestrating processes that are important for wound healing. Neutrophils are responsible for clearing debris and pathogens, macrophages for guiding tissue repair by dynamic M1 to M2 polarization, and T cells for regulating adaptive immunity. Upon injury of the skin, transforming growth factor-beta (TGF-β), a multifunctional cytokine implicated in the regulation of inflammation and tissue regeneration, is released by platelets to recruit neutrophils. Afterward, the macrophages change from the pro-inflammatory M1 phenotype to the reparative M2 phenotype, while fibroblasts participate in ECM reconstruction 39-41. Macrophages release the growth factors, VEGF and platelet-derived growth factor, which play a key role in angiogenesis and epithelial regeneration.

In the case of diabetic wounds, this finely tuned repair program is broken. Hyperglycemia inhibits M2 macrophage polarization through ROS/NF- κB signaling axis, which sustains a predominance of the pro-inflammatory M1 phenotype and perpetuates chronic inflammation 42-45. Additionally, the ECM altered by advanced glycation end products (AGEs) negatively affects healing by impairing TGF-β signaling, blocking fibroblast migration and reducing the response of endothelial cells to the growth factor VEG during the inflammatory phase. Emerging therapeutic approaches are aimed at "restarting" the impaired repair process. These include delivery of anti-inflammatory cytokines such as IL-10 or TGF-β using nanocarriers to induce M2 macrophage activation, as well as modulation of ROS/NF- κB signaling pathways using MOFs. Tumor necrosis factor-stimulated gene-6 (TSG-6), an anti-inflammatory glycoprotein released by mesenchymal stem cells, plays a very important role in immune balance restoration and ECM remodeling, acting as a key mediator in the use of stem cell-based therapies in the reshaping of the immune niche 44-50. Functional hydrogels also play a role by relieving hypoxia and improving angiogenesis. Collectively, these strategies address the intertwined triad of inflammation, hypoxia, and infection that is the basis for impaired diabetic wound healing.

#### 2.2.1 Inflammation phase

**Figure 2:**
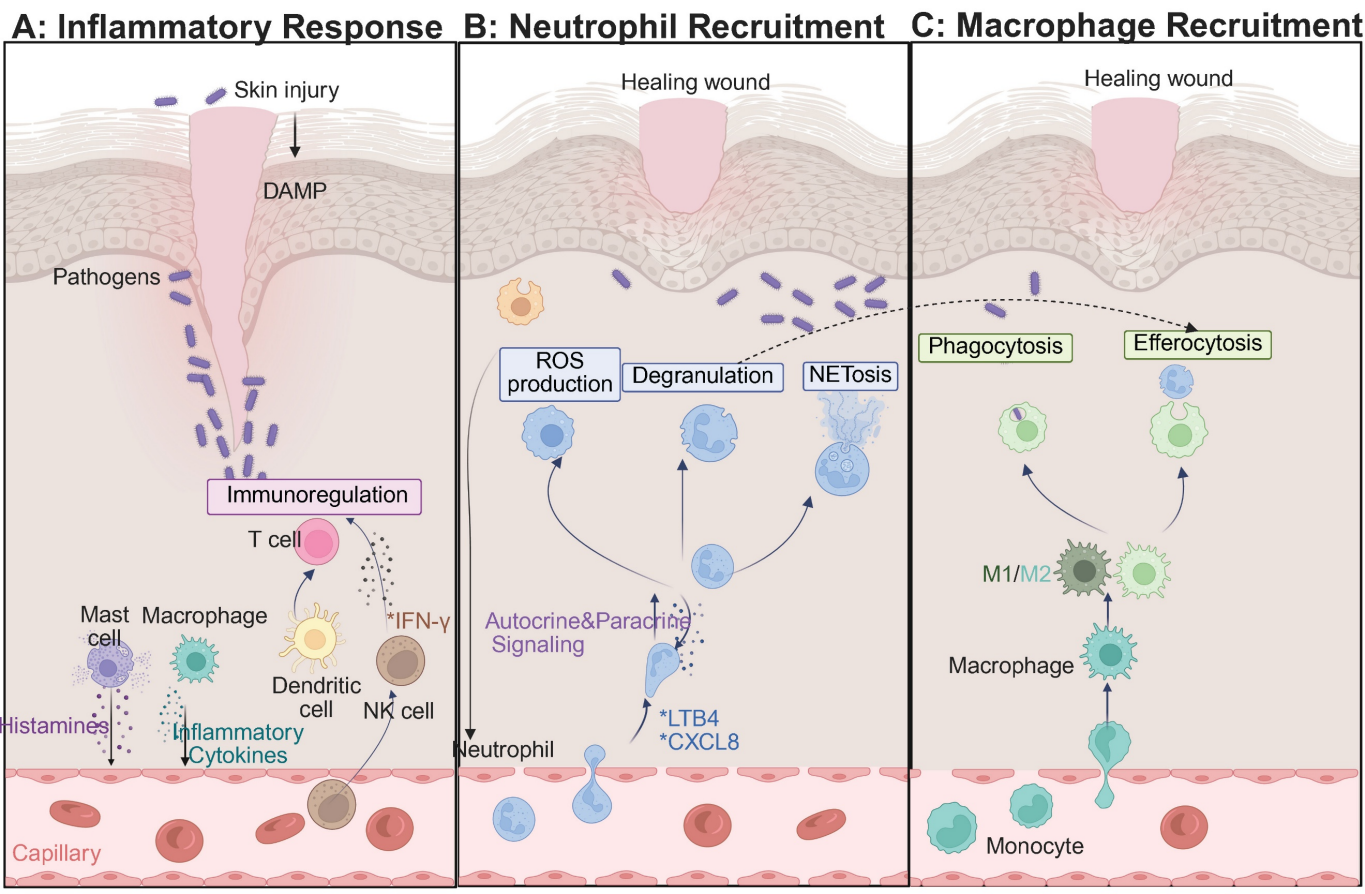
Dynamic evolution of immune microenvironment in the inflammatory phase of diabetic wound healing (A: inflammatory; B: neutrophil recruitment; C: macrophage recruitment). Created in BioRender. Jia, J. (2025) https://BioRender.com/lcu92wa.

Hyperglycemia disrupts the skin barrier, promotes bacterial colonization, and increases inflammatory responses. Under high glucose conditions, mitochondrial dysfunction and activation of the enzyme NADPH oxidase cause increased ROS production, which causes damage to vascular endothelial cells and collagen, and inhibits macrophage polarization toward the anti-inflammatory M2 phenotype and secretion of IL-10 and TGF-β 51-54. This metabolic imbalance traps macrophages in the pro-inflammatory state called M1, and creates a vicious cycle of inflammation, oxidative stress, and infection. Diabetic wounds are associated with significant immune dysfunction, including a M1/M2 macrophage ratio more than three-fold higher than in healthy tissue, elevated levels of pro-inflammatory cytokines IL-6 and IL-12, impaired angiogenesis, and excessive neutrophil extracellular traps (NETs), DNA-protein complexes secreted by neutrophils that trap microbes but also contribute to persistent tissue damage 55-59. Additionally, T cells and dendritic cells become skewed to pro-inflammatory phenotypes accompanied by decreases of regulatory T cells (Tregs) and Th2 cells as well as increases in chemokine expression that inhibit fibroblast and endothelial cell functions 60-62. Targeted therapeutic interventions aimed at promoting M2 macrophage polarization, collagen synthesis and re-epithelization are showing considerable promise. Engineered hydrogels and nanomaterials carrying IL-4, IL-13 or small interfering RNA (siRNA), which induces sequence-specific gene silencing through RNA interference, have shown promise in the modulation of the wound immune microenvironment 63-65 (**Figure 2**). Future research should be aimed at understanding the complex interactions between immune and matrix cells in order to develop precision strategies for chronic wound repair.

#### 2.2.2 Proliferation phase

**Figure 3:**
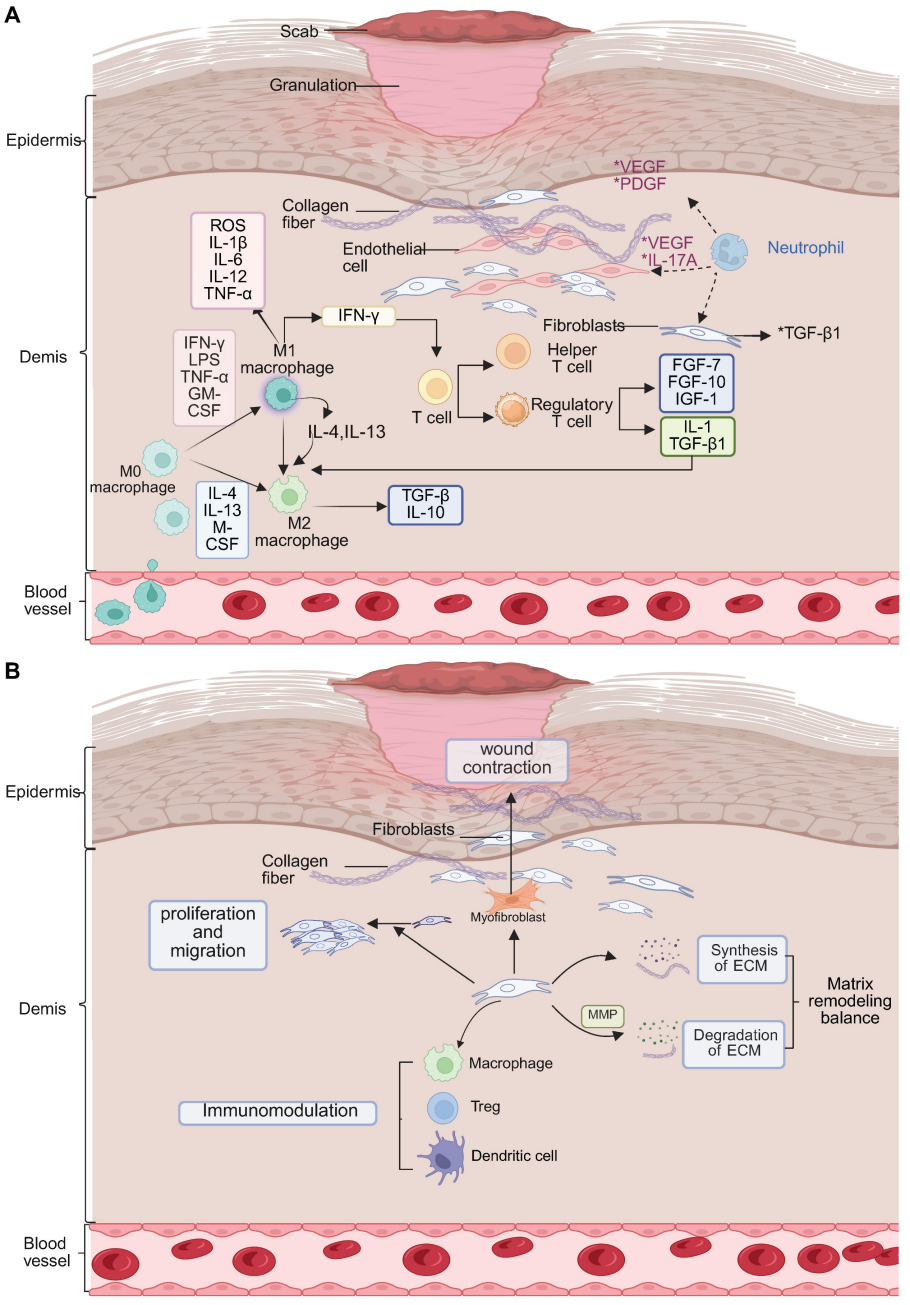
(**A**) Dynamic evolution of immune microenvironment in the proliferative phase and (**B**) Reshaping phase of diabetic wound healing. Created in BioRender. Jia, J. (2025) https://BioRender.com/f8nmo43.

During the proliferative phase of diabetic wound healing, the regulation of the immune system is severely affected by hyperglycemia. Unlike the normal healing process, which promotes faster resolution by apoptosis of neutrophils and the activity of pro-resolving mediators, AGEs interact with their receptor (RAGE) in diabetic wounds, inhibiting macrophage autophagy by ~60%, which delays the important switch between the pro-inflammatory M1 and the reparative M2 phenotype 66, 67. This impairment results in delayed clearance of cellular debris, increased ROS generation, impaired dendritic cell antigen presentation, and diminished phagocytic function of neutrophils and together leads to disruption of immune homeostasis. Normally, M2 macrophages make up some 70% of the immune cell population in wound tissue. However, there is an association between diabetes and a higher number of Treg cells and a reduced number of functional NK cells, which contribute to suppressed immune surveillance 68-70. Pro-inflammatory Th17 and γδ T cells further increase the inflammation, and dendritic cells are unable to sufficiently support epidermal proliferation (**Figure 3A**). The polarization of M2 macrophages mediated by IL-4/IL-13-STAT6 signaling, adenosine-A2a receptor activation and miR-21-5p/PI3K-Akt pathway is suppressed under hyperglycemic conditions 71, 72. Impaired clearance of NETs leads to oxidative stress "storms" that result in damage to the endothelium. Therapeutic interventions include IL-10 loaded hydrogels which have shown enhanced healing effects 73. Toll-like receptor 7/8 agonists have been shown to induce M2 macrophage polarization, and peptidylarginine deaminase 4 inhibitors inhibit ROS accumulation. In addition, Sema4D-miR-21 scaffolds are also being investigated for their angiogenic potential 74. Gene editing on RORγt and PD-1 pathways is a promising approach to precise immune modulation 75-77. Ultimately, the translation of these findings into clinical practice requires a multifaceted approach to harmonize resolution and regeneration through spatiotemporal, multi-omics-based interventions.

#### 2.2.3 Reshaping phase

In the remodeling phase of diabetic wound healing, AGEs have been shown to destroy collagen cross-linking by covalent modification, leading to a reduction in tensile strength of more than 60% 11, 78. Concurrently, the ratio of MMP-9 to its tissue inhibitor TIMP-1 changes dramatically to 5:1, favoring excessive and disorganized degradation of ECMs. Macrophage-derived lysyl oxidase (LOX), an extracellular enzyme important in the crosslinking of collagen and elastin, shows a 40% reduction in activity 79, 80. The HIF-1α-mediated glycolytic pathway favors pro-inflammatory M1 macrophages over M2 phenotypes, disrupting fibroblast contraction through elevated IL-6 and TNF-α levels and inhibiting apoptosis of myofibroblasts 81. Treatment with metformin, which acts through AMP-activated protein kinase, has been seen to restore M2 macrophage polarization by 50%, and normalizes LOX activity by 70%. Neutrophils have a significantly extended half-life, ~12-fold, and contribute to chronic NETosis and M2c macrophage polarization via histone H3 signaling. DNase I therapy has been shown to decrease the thickness of scars by 50% 82, 83. Additionally, apoptosis of Tregs cells and IL-10 deficiency cause impairment in antifibrotic functions, while NK cells have a 50% impairment in their ability to clear senescent fibroblasts due to NKG2D glycosylation alterations 84-86. The pathological basis of diabetic wound remodeling thus involves disruption of the AGEs-RAGE-MMP-9 axis, dysfunction in metabolism-mechanics signaling, and hypoxia marked by a 40% reduction in blood flow. Therapeutic interventions such as HIF-1α inhibitors, pH-responsive DNase I, and copper oxide (CuO)-loaded biomimetic scaffolds have shown promise, enhancing healing rates by twofold (**Figure 3B**) 87, 88. Multiscale regulation of the interconnected “matrix-immunity-metabolism” triad is essential for reversing chronic fibrosis and promoting effective wound remodeling.

## 3. Types of Microenvironment-Responsive Hydrogels

Chronic diabetic wounds constitute a highly dynamic and pathologically complex microenvironment, often characterized by persistent hyperglycemia, chronic inflammation, excessive oxidative stress and abnormal MMP activity all of which severely impair tissue regeneration 89, 90. Although conventional hydrogel dressings offer advantages such as moisture retention and physical protection, they are usually not able to actively sense and respond to pathological cues. As a result, they fail to meet the stage-specific and multi-target and changing needs in the management of a diabetic wound 25, 90-92. In this regard, microenvironment-responsive hydrogels have become an advanced class of functional biomaterials that can sense pathological stimuli, such as glucose, pH, ROS, and MMPs, and then induce spatiotemporally controlled drug release and wound microenvironment modulation. By adding dynamic covalent bonds, enzyme sensitive peptide linkers or redox responsive moieties, these hydrogels exhibit smart responsiveness, which allows for simultaneous control of inflammation, stimulation of angiogenesis, and decrease of oxidative damage 93-100. Based on their stimulus recognition mechanism, these systems are broadly categorized as glucose-responsive, pH-responsive, enzyme-responsive and ROS-responsive hydrogels 101-104. The following sections systematically review their design principles, therapeutic mechanisms, and recent advancements in the remodeling of the diabetic wound microenvironment to give both theoretical insight and practical guidance for the development of next-generation multi-responsive platforms.

### 3.1 Glucose-responsive hydrogel

Chronic diabetic wounds are closely associated with the hyperglycemic microenvironment. Prolonged hyperglycemia not only provides a nutrient-rich substrate for bacterial proliferation, which increases the infection risk of pathogens such as Staphylococcus aureus and Pseudomonas aeruginosa by over threefold 105, but also persistently activates the M1 polarization of macrophages. This activation results in excessive release of proinflammatory cytokines (e.g. IL-6, TNF-α) and increased oxidative stress 106. Additionally, deposition of AGEs inhibits the expression of VEGF, leading to tissue hypoxia and failure of vascular regeneration. Together these factors form a vicious cycle of hyperglycemia, infection, inflammation, and vascular damage 107.

**Figure 4:**
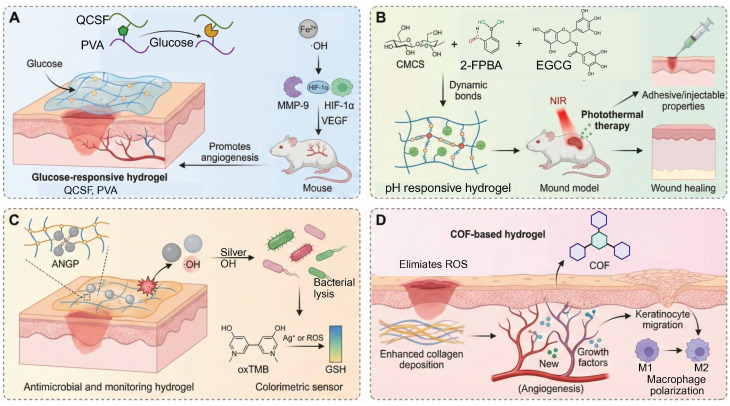
Types of microenvironmental responsive hydrogel. (**A**) Glucose-responsive hydrogel. (**B**) pH-responsive hydrogel. (**C**) Enzyme-responsive hydrogel. (**D**) ROS-responsive hydrogel. Created in BioRender. Jia, J. (2025) https://BioRender.com/4spcrm7.

In order to break this harmful cycle, researchers have developed smart hydrogel systems based on multi-responsive mechanisms. Among these, catalytic systems driven by glucose oxidase (GOx) have shown excellent but condition-dependent performance. For example, a boronic ester-crosslinked hydrogel based on modified quaternized chitosan and polyvinyl alcohol (PVA), which reacts with high glucose levels and hydrogen peroxide (H_2_O_2_), breaks dynamic boronic ester bonds, thus reducing oxidative stress and releasing deferoxamine-loaded gelatin microspheres (DFO@G). The activation level for this glucose triggered reaction (≈15 mM) is very close to the pathological blood glucose range found in diabetic wounds (10-25 mM), ensuring physiological relevance. Subsequently, MMPs promote the long-term release of DFO, which promotes the expression of HIF-1α and angiogenesis, promoting the healing of diabetic wounds (**Figure 4A**) 101. More innovative self-regulating systems, such as MN-GOx/Arg microneedles, are responsive to MMP-9 overexpression in the wound microenvironment and trigger arginine release via cascade reactions. This facilitates collagen deposition and angiogenesis which enhances the wound healing rate in diabetic rats by 2.3 times 108, 109. Similarly, Li *et al.* developed a quaternized chitosan/salvianolic acid B (QF/SAB) hydrogel with dynamic boronic ester and imine bonds, which are sensitive to both glucose and ROS. This allows on-demand drug release, ROS scavenging, and improved angiogenesis with more than 90% wound closure in diabetic models 110. A triple-responsive hydrogel that quickly releases insulin in the acidic (pH 5.5-6.5) and high temperature (40-42 °C) environments characteristic of wound surfaces through the synergistic effect of the Schiff base, boronic acid ester and hydrogen bond interactions. In addition, it has a photothermal antibacterial effect and inhibits over 95% of methicillin-resistant Staphylococcus aureus (MRSA) under near-infrared (NIR) irradiation 111. Photoresponsive hydrogels are a new method of deep-tissue repair. For example, a Rh/AgMoO nanozyme composite hydrogel produces ROS (•OH and O₂⁻) and hydrogen under visible light excitation. This process inhibits bacterial glycolysis by disrupting the phosphotransferase system and downregulates the expression of the RAGE improving the wound healing rate of diabetic mice by 78% 112. Experiments have demonstrated that such hydrogels can simultaneously control macrophage M2 polarization and promote angiogenesis to achieve multidimensional microenvironmental repair. Comparative studies have shown that glucose responsive systems are more specific for their substrates than pH or ROS responsive hydrogels, but are more sensitive to variations in oxygen concentration and enzyme stability. Despite these promising results, the clinical translation of glucose-responsive hydrogels is faced with several challenges. The stability and catalytic efficiency of GOx are affected by variations in pH and local hypoxia conditions, which results in inconsistent performance *in vivo*. Moreover, the overaccumulative byproduct of the oxidation of glucose, H_2_O_2_, may cause oxidative stress and tissue irritation. Future studies should be designed to combine biomimetic enzyme mimics and oxygen-controlled cascade systems to enhance long-term catalytic efficiency, biosafety, and clinical reliability.

### 3.2 pH-responsive hydrogels

The pH of diabetic wounds is not static but changes dynamically throughout the healing process. The wound environment during the inflammatory stage is generally mildly alkaline, which is thought to be due to chronic exudation and increased protease activity 113. As infection progresses, the metabolism of bacteria and tissue necrosis lead to the accumulation of lactic and fatty acids, which creates a microenvironment that is more acidic 114. These dynamic pH changes represent the pathological changes that occur at various stages of healing in chronic, non-healing diabetic wounds, underscoring the complex interplay between inflammation, tissue necrosis and bacterial colonization 115. Accordingly, the design of pH-responsive hydrogels that can adapt to these fluctuations is crucial in order to ensure efficient drug delivery during the healing process. Such responsiveness is mainly accomplished by reversible ionization and or dynamic covalent bonding mechanisms that are sensitive to pH variations.

In material design, natural polysaccharides like chitosan and gelatin are commonly used because of their good biocompatibility and cell proliferation. For example, modified gelatin/chitosan composite systems significantly increase the pH responsiveness and antibacterial activity under laboratory conditions due to dopamine coating and quaternization treatments 116. On the other hand, synthetic polymers such as PVA and polyacrylic acid (PAA) are frequently used. PVA/CS-BA systems that are formed by dynamic covalent cross-linking, e.g., borate and hydrogen bonds, can intelligently release pro-angiogenic drugs in response to the acidic environment induced by infection and metabolic byproducts such as lactic acid, while retaining a moist wound environment 117. This pH-triggered drug release, which is effective in a pH range of 5.5 to 7.0, is quite compatible with the pH variations that typically occur in diabetic wounds (pH 5.4-8.9), thus highlighting its physiological relevance 118. Recent studies highlight that the pH-dependent assembly or disassembly of the phytochemical-based hydrogels based on tannic acid, gallic acid, and epigallocatechin gallate (EGCG) occurs via hydrogen bonding and π-π stacking. This allows acidity induced softening and accelerated release of intrinsic bioactive components without need for additional carriers 119. Notably, a recently developed EGCG-based adhesive hydrogel based on carboxymethyl chitosan and 2-formylphenylboronic acid via dual dynamic covalent bonds with favorable mechanical strength, antioxidant capacity, and pH-controlled EGCG release. This design also provides on-demand dissolvability to reduce secondary damage during dressing removal (**Figure 4B**) 102.

Furthermore, multi-component composite hydrogels incorporating PAA and natural cellulose along with modified carbon quantum dots, exhibit both self-healing properties and real-time pH monitoring 120. When wound pH is reduced from 7.4 to 5.8 by bacterial infection, carbon quantum dots induce controlled drug release, and antimicrobial peptides release rate is 3.8-fold higher. Simultaneously, the addition of zinc oxide nanoparticles results in improved mechanical properties (compressive strength up to 45 kPa) as well as antioxidant efficiency (the 2,2-diphenyl-1-picrylhydrazyl radical scavenging rate is more than 90%). This pH-responsive and graded release mechanism not only enhances insulin release in acidic conditions (4.2-fold increase in release rate) but also burst release of antimicrobial drugs in alkaline conditions, which reduces inflammatory factors and enhances the process of angiogenesis. Moreover, phytochemical alkaloids such as berberine and flavonoids such as curcumin show increased solubility and intermolecular mobility in mildly acidic microenvironments, which allows self-controlled release kinetics to complement conventional polymeric pH-responsive platforms.

In summary, pH-responsive hydrogels, with their ability to perform precise drug delivery, intelligent monitoring, and multifunctional integration, are a versatile platform for the management of diabetic wounds. However, the dynamic pH range in chronic wounds is relatively small and spatially heterogeneous, which often restricts the full activation of these systems. Localized infection and necrosis can create conflicting micro-pH gradients and buffering capacity of the wound exudate may further reduce responsiveness. Compared to glucose or enzyme responsive systems, pH responsive hydrogels provide a faster but less specific regulatory response. These limitations indicate that future designs should combine pH sensitivity and biochemical recognition mechanisms to provide more precise and sustained therapeutic control.

### 3.3 Enzyme-responsive hydrogels

In human tissues, enzymes are ubiquitous natural biocatalysts. Their high specificity and rapid response enable them to be known to play key roles in ECM remodeling, cell migration, and signal transduction 29, 121. During normal wound healing, enzymes participate in inflammation regulation, angiogenesis, and collagen deposition, thereby promoting tissue regeneration. However, due to persistent hyperglycemia and oxidative stress, the local microenvironment of diabetic wounds is significantly disturbed, accompanied by chronic inflammation and abnormally elevated MMPs and cyclooxygenase-2 activities^122^. These abnormal enzymes not only disrupt the normal secretion of growth factors such as VEGF and EGF, but also inhibit the formation of granulation tissue by excessively degrading ECM components such as collagen, ultimately leading to stagnation of wound repair. In addition, iron ion overload and ROS accumulation further aggravate tissue damage forming a vicious cycle 123.

Enzyme-responsive hydrogels have a three-dimensional network structure similar to that of ECM, and are self-repairing and highly permeable, and can dynamically respond to the complex microenvironment of diabetic wounds 31, 108, 124. A POD-mimetic lysozyme-hemin nanozyme-anchored Gelatin/PVA hydrogel (ANGP) exploits bacterial GSH-mediated catalysis for rapid colorimetric detection and •OH-based sterilization, though long-term hemin release remains to be optimized (**Figure 4C**) 103. Compared with traditional dressings that passively release drugs, this type of hydrogel responds to abnormal enzyme activity through dynamic covalent bonds (such as phenylboronic acid ester bonds) or breakable connections composed of MMP-sensitive peptides, thereby achieving precise on-demand release of drugs and regulation of the microenvironment 125. For example, in wound areas where MMP-9 is overexpressed, hydrogels can simultaneously release anti-inflammatory drugs or iron chelators after enzymatic degradation, maintaining the structural stability of the ECM while exerting anti-inflammatory and oxidative stress-reducing effects 126. This dual function gives it significant advantages in scavenging ROS, inhibiting inflammation, and promoting angiogenesis.

In response to the challenges of MMP overexpression and iron ion imbalance in diabetic wounds, researchers designed a composite hydrogel system based on modified alginate and polyethylene glycol diacrylate (PEGDA). This system introduces MMP-sensitive peptides as crosslinkers and loads microspheres containing the iron chelator deferoxamine (DFO) to achieve a dual response to abnormal enzyme activity and iron ion overload 126. In an acidic environment or in areas with high MMP expression, the hydrogel releases DFO through enzymatic degradation, effectively chelating free iron, reducing ROS levels, improving the microenvironment, and promoting vascular endothelial cell migration. Animal experiments have shown that this system was reported to increase the expression of VEGF and CD31 and to promote collagen deposition and granulation tissue formation, and thus shortens the healing cycle.

In order to overcome the problems of low drug release efficiency and insufficient biological activity of traditional hydrogels, researchers further integrated mesenchymal stem cell-derived Exos (MSC-exos) into enzyme-responsive hydrogels. For example, the "MSC-exo@MMP-PEGDA" hydrogel embeds Exos into the MMP-sensitive cross-linking network through electrostatic adsorption 127, 128. In the area where MMP-9 is highly expressed, the hydrogel gradually degrades and continuously releases Exos. Exos can activate the PI3K/Akt/eNOS signaling pathway to promote endothelial cell proliferation, while inhibiting NF-κB-mediated inflammatory response. The miRNAs they carry can also regulate ECM synthesis-related genes, reduce excessive collagen deposition and inhibit scar formation. This composite system showed a sustained release effect of up to four weeks in a diabetic rat model, significantly improving re-epithelialization and angiogenesis.

In summary, enzyme-responsive hydrogels have unique merits in the controlled drug release and adaptive microenvironment regulation via biologically specific degradation 108, 129. However, their performance remains highly dependent on the fluctuating enzyme activities present in chronic diabetic wounds, which can result in inconsistent release profiles. The synthesis of enzyme-cleavable linkers and incorporation of bioactive cargos also increase manufacturing complexity and limit large-scale production. Compared with pH- or ROS-responsive systems, enzyme-responsive hydrogels exhibit superior specificity but slower response kinetics and reduced structural stability 12, 113, 130. Therefore, future research should focus on designing hybrid hydrogels that integrate enzyme-responsive degradation with other stimuli-sensing elements to enhance precision and translational feasibility.

### 3.4 ROS-responsive hydrogel

ROS are a class of highly oxidative molecules, including O_2_^-^, H₂O₂ and ·OH, which are inevitable byproducts of cellular metabolism. Diabetic wounds fall into a vicious cycle of "difficult to heal-infected-necrotic" due to the imbalance of oxidative stress caused by persistent hyperglycemia. ROS have a biphasic regulatory effect in wound repair. At physiological concentrations, ROS promote angiogenesis by activating the VEGF signaling pathway and drive the bactericidal function of neutrophils 131. However, in the diabetic wound microenvironment, abnormal mitochondrial electron transport chain leads to a surge in ROS (>100 μM H₂O₂), breaking through the redox homeostasis threshold, triggering overactivation of the NF-κB signaling pathway, prompting macrophages to polarize to the pro-inflammatory M1 type, inhibiting collagen deposition and exacerbating ECM degradation 25, 132. Studies have shown that ROS levels in diabetic wounds are 3-5 times higher than those in normal tissues, forming a positive feedback loop with pro-inflammatory factors such as TNF-α and IL-6, further aggravating hypoxia and vascular regeneration disorders 133. Although traditional hydrogel dressings can provide a physical barrier and local moisturizing, they lack dynamic response capabilities: single antioxidant components (such as vitamin C) are easily exhausted in the early stage and cannot cope with continuous ROS generation; the separation of antibacterial and healing functions leads to insufficient control of deep tissue infection, and the hypoxic microenvironment hinders late epithelial regeneration 134. For example, although ordinary chitosan hydrogels have antibacterial properties, they cannot target the removal of intracellular ROS or regulate macrophage phenotypic transformation 135.

In view of the limitations of traditional materials, smart responsive hydrogels have attracted increasing attention due to their dynamic molecular design and technological integration. Among them, the “ROS threshold trigger” mechanism based on dynamic covalent bonds has emerged as a key approach: the thioketone bond (-SS-) and the phenylboronic acid bond are selectively broken under pathological ROS levels(Among them, the -SS- and the phenylboronic acid bond are selectively broken under pathological ROS levels (typically ≈100 μM H₂O₂, within the physiological ROS range of 50-200 μM in diabetic wounds)) to achieve spatiotemporal controllable drug release. For example, CMCS-PBA/OXD hydrogel preferentially releases α-lipoic acid in the high ROS area of the wound through ROS-sensitive Schiff base bonds to modulate ROS levels and inhibit biofilm formation of Staphylococcus aureus 12. In addition to the ROS responsive dynamic linkages commonly used in synthetic hydrogels, several traditional Chinese medicine (TCM) derived phytochemicals such as baicalein, quercetin, and resveratrol have been reported to undergo ROS mediated oxidation or structural softening 136-142. This behavior increases molecular mobility and promotes the release of their intrinsic antioxidant components. These TCM related antioxidant frameworks thus offer a biodegradable and biocompatible supplementary mechanism for ROS responsive modulation in chronic diabetic wounds, while avoiding the long term biosafety issues associated with metal based catalytic systems. For the improvement of the hypoxic microenvironment, the bionic cascade strategy of oxygen self-supply and antioxidant system shows significant advantages. For instance, a dynamic oxygen-releasing hydrogel constructed by encapsulating basic fibroblast growth factor (bFGF), microalgae, and COF-1 into a self-assembled network demonstrates synergistic effects in alleviating oxidative stress and promoting angiogenesis. The borate-based covalent organic framework (COF) achieves ROS scavenging and glucose-responsive degradation, while the microalgae continuously provide the dissolved oxygen to alleviate the hypoxia. In addition, the B-N coordination bonds assist in pH-sensitive drug release. This multifunctional hydrogel has a favorable biocompatibility and can promote re-epithelization and collagen remodeling in diabetic wounds, which is a promising strategy for wound management in chronic wounds (**Figure 4D**) 104.

In summary, ROS-responsive hydrogels can effectively modulate oxidative stress, angiogenesis enhancement, and immune remodeling via threshold-triggered and cascade catalytic mechanisms. However, the concentration and distribution of ROS in chronic wounds is highly variable and this makes precise activation control difficult. Excessive scavenging of ROS may impair normal redox signaling, whereas insufficient response could fail to alleviate oxidative damage. In addition, the long-term biosafety of metal-based nanozymes and quantum dots remains uncertain, and their mechanical stability is often inadequate for deep wound support. Compared with enzyme- or glucose-responsive systems, ROS-responsive hydrogels provide faster responses but lower selectivity and reduced structural integrity. Therefore, future research should aim to establish quantitative redox regulation and develop biodegradable, metal-free catalytic systems to achieve safe, tunable, and clinically reliable wound therapies.

To provide a concise summary of the single-stimulus systems discussed above,** Table 1** compiles representative examples of glucose-, pH-, enzyme-, and ROS-responsive hydrogels reported in 2024-2025, along with their key physical parameters. This comparative overview highlights how each stimulus type influences the mechanical, adhesive, and degradation behaviors of the hydrogels, offering a foundation for the development of multi-responsive platforms discussed in the next section.

As summarized in **Table 1**, single-stimulus microenvironment-responsive hydrogels show different physical behaviors mainly due to the differences in their molecular crosslinking mechanisms. Glucose- and enzyme-responsive systems typically employ covalent or enzyme cleavable linkages that yield denser polymer networks, providing higher mechanical strength and slower and more controlled rates of degradation. In contrast, pH- and ROS-responsive hydrogels are typically formed by reversible dynamic bonds, such as imine, borate, or disulfide bonds, that are easily cleaved under acidic or oxidative conditions. These structural characteristics encourage higher network relaxation, swelling ratios, and faster disassembly, which aids in on-demand release of drugs.

Overall, these distinctions emphasize the specific benefits and drawbacks of each stimulus type in striking the right balance between stability and responsiveness. Such insights offer a structural basis for the design of multi-responsive hydrogels with complementary properties, which will be discussed in the next section.

### 3.6 Multi-responsive hydrogels

**Figure 5:**
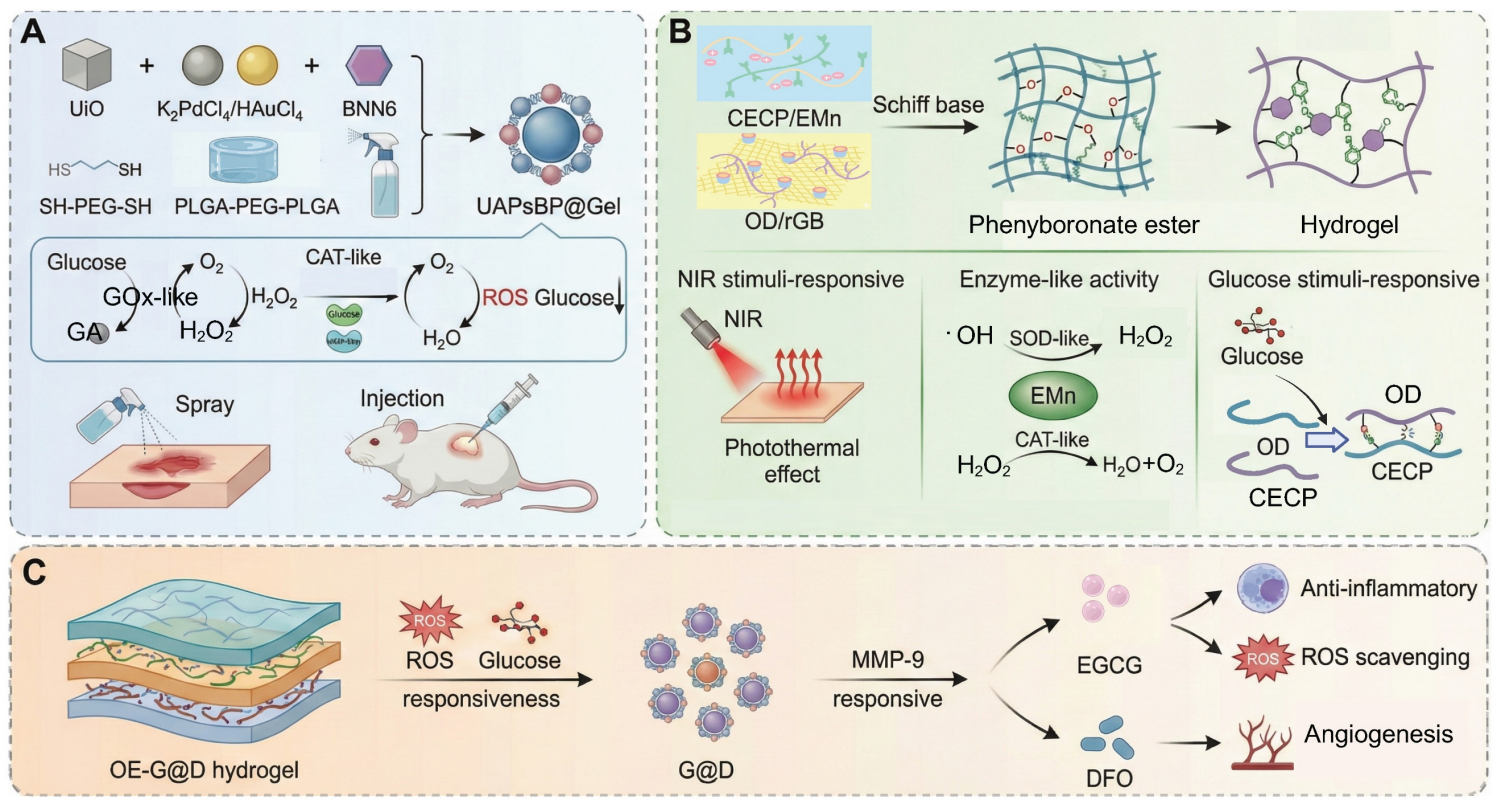
Types of multi-responsive hydrogel. (**A**) pH/ROS responsive hydrogel. (**B**) NIR/Glu/ROS responsive hydrogel. (**C**) ROS/Glu/MMP responsive hydrogel. Created in BioRender. Jia, J. (2025) https://BioRender.com/f9fv3g2.

Diabetic wounds are defined by a vicious cycle, including hyperglycemic microenvironment, constant oxidative stress (ROS), overexpression of MMP-9 and the imbalance of inflammatory mediators (especially the IL-10/TNF-α ratio). This complex and dynamically intertwined pathology poses great challenges to conventional single-responsive hydrogels. For instance, strategy that is focused only on scavenging ROS may result in excessive ROS depletion, impairing their physiological pro-angiogenic functions. Similarly, a hydrogel that can only respond to pH changes has difficulty responding to the multi-stage requirements of diabetic wounds, which include anti-infection, anti-inflammation, and tissue regeneration 148. In this context, multi-stimulus responsive systems are on the verge of integrating signals of glucose, ROS, pH, enzymatic activity and temperature to achieve temporally and spatially adaptive, precisely synergistic therapeutic effects. During the first inflammatory phase, a dual pH/ROS-responsive module can release antimicrobial agents to inhibit infection (**Figure 5A**) 143. In the following phase, the regulation of redox balance and immune microenvironment is accomplished by the action of enzymes or temperature-sensitive mechanisms. Finally, in the remodeling phase, the sensitivity of glucose may be used to stimulate angiogenesis and the ECM remodeling process to aid in the entire wound healing process 121. This development of dynamic sensing and multi-modal responsiveness highlights the potential of multi-responsive hydrogels as a transformative technology to overcome the persistent challenges in diabetic wound healing.

To meet the complex requirements in diabetic wound treatment, a hierarchical and progressive design paradigm has been proposed by researchers 144-151. For instance, a COF-modified microalgae hydrogel system was created that combines glucose, ROS and NIR light responsiveness in one therapeutic system 144. The boronic ester-based COF-1 scavenges ROS and allows glucose-triggered release of bFGF and embedded microalgae continuously generate oxygen to relieve hypoxia (**Figure 5B**). Upon 808 nm NIR irradiation, localized heating promotes the permeability of the gel and increases the speed of cargo release. This synergistic system enhances angiogenesis, inhibits inflammation and restores redox balance, which greatly enhances diabetic wound closure and tissue regeneration through a cascade-responsive mechanism. The combination of boronic ester-linked COFs and microalgae enables glucose- and ROS-responsive drug release, and NIR-induced oxygenation. This spatiotemporal synergy counteracts oxidative damage caused by hyperglycemia and re-establishes redox homeostasis, promoting angiogenesis, improving cell proliferation and accelerating chronic diabetic wound repair without interfering with physiological ROS signaling. Another innovative approach is through the adaptation of the healing process through a chronological sequential release strategy (**Figure 5C**). For example, a glucose/ROS/MMP-9 multi-responsive hydrogel (OE-G@D system) is sensitive to hyperglycemia in the early stage of inflammation by breaking phenylboronate bonds to release EGCG, which scavenges ROS and inhibits the accumulation of AGEs. This is followed by MMP-9-mediated release of DFO to stimulate angiogenesis 145. During the repair phase, dissolution of a polysaccharide called chitosan under acidic conditions releases manganese-based nano-enzymes, which improves ROS scavenging efficiency and the rate of collagen deposition is increased by 50%. Cutting edge research is also being conducted on adaptive closed-loop systems, such as a dual-responsive electric field/temperature dressing that activates precise insulin delivery via a polyaniline conductive network that senses changes in wound potential (with sensitivity of 0.1 mV/mm^2^). This system integrates coupling temperature-sensitive polymer segments to mediate controlled release of VEGF, in addition to real-time biomarker monitoring using flexible microelectrodes, to create a dynamic "sense-respond-regulate" closed-loop treatment platform 147.

Despite the great progress, clinical translation of multi-stimulus-responsive hydrogels is still hindered by three key challenges: (1) material incompatibility, e.g. the tendency of uncontrolled cross-linking between ROS-sensitive thioether bonds and enzyme-sensitive peptide chains 148, 149; (2) poor dynamic adaptability, i.e. delayed response to rapid pH fluctuation or pulsed bursts of ROS 150; and (3) heterogeneity in large-scale production, such as AuNRs/chitosan complexes, which are suffering from batch-to-batch variability due to nanoparticle aggregation 151. Specifically, chitosan-based complexes are prone to inconsistencies due to clustering of nanoparticles which affects reproducibility. To overcome these obstacles, future research is increasingly focusing on interdisciplinary and synergistic innovations. These include programming biomimetic topologies using DNA origami technology to build smart materials that can dynamically respond to multiple biomarkers such as MMP-9, IL-6 and VEGF; using genetically engineered cyanobacteria implanted into wounds to achieve simultaneous photosynthetic oxygenation and necrotic tissue degradation; and developing 3D-printed gradient drug-release hydrogels that incorporate macrogenomic data of the patients microbiota for personalized microbial modulation 152-155.

## 4. Designation of Cascade Reaction in the Responsive Hydrogels Combine with Treatment of Diabetic Wounds

Diabetic wound healing is a complex pathophysiological network, where the main difficulty of wound healing is caused by the spatiotemporal interaction of several pathological factors, such as bursts of ROS, protease imbalances, chronic inflammation, and impaired vascular regeneration in a hyperglycemic microenvironment. Traditional single target therapies have difficulty interrupting this multidimensional vicious cycle because of limited targets and mismatched therapeutic time windows. In recent years, cascade reaction strategies have been able to achieve temporal adaptation of multi-target synergistic intervention and therapeutic effects through the use of a trigger-response-amplification regulatory logic. Smart responsive hydrogels are suitable carriers for these strategies, allowing controlled delivery of cascade signals induced by pathological microenvironmental cues (such as pH, ROS levels or enzyme activity) to reshape the regenerative microenvironment in both spatial and temporal dimensions 12, 155. Building on this concept, combined therapies not only improve the accuracy of the treatment, but also increase the biological effect of cascade reactions. Examples include the ability to simultaneously control angiogenesis and anti-inflammatory response through cascade release of nitric oxide (NO), or the use of cascade enzymatic reactions to selectively target and destroy excessive inflammatory mediators 19. To systematically explore these mechanisms, this study uses a three-tier classification system according to reaction chain length, molecular mechanism, and treatment stage targeting to give a comprehensive analysis of the adaptation logic and translational potential of cascade reaction strategies for diabetic wound therapy 156.

### 4.1 Classification based on the number of cascade reactions

Given the complex pathological characteristics of diabetic wounds, including persistence of hyperglycemic microenvironment, drug-resistant bacterial infections, oxidative stress and inflammatory storms, traditional monotherapies often struggle to achieve effective dynamic intervention. In recent years, cascade-based stimulus-responsive hydrogel systems have become a cutting-edge approach to address these multifaceted challenges by a hierarchical and progressive treatment logic.

To gain a better understanding from a conceptual point of view, we use a more rigorous definition of a true cascade reaction: one in which the intermediate or product formed at one step directly drives or catalyzes the next step, leading to a self-propagating or sequentially amplified reaction. By contrast, systems which are triggered by a single stimulus are categorized as single-stimulus-responsive systems and do not, strictly speaking, constitute cascade reactions.

Accordingly, the systems reviewed in this section are classified according to the number of cascade levels, i.e. single-stimulus responsive systems, dual-layer cascades, and multi-layer cascade systems (**Table 2**). This classification reflects the different levels of coupling of reactions and functional complexity. It is important to emphasize that this framework is conceptual rather than a strict chemical-step definition and is used to clearly distinguish between simple stimulus-response systems and true multistep cascades while retaining internal consistency in the current discussion.

#### 4.1.1 Single-stimulus responsive system

**Figure 6:**
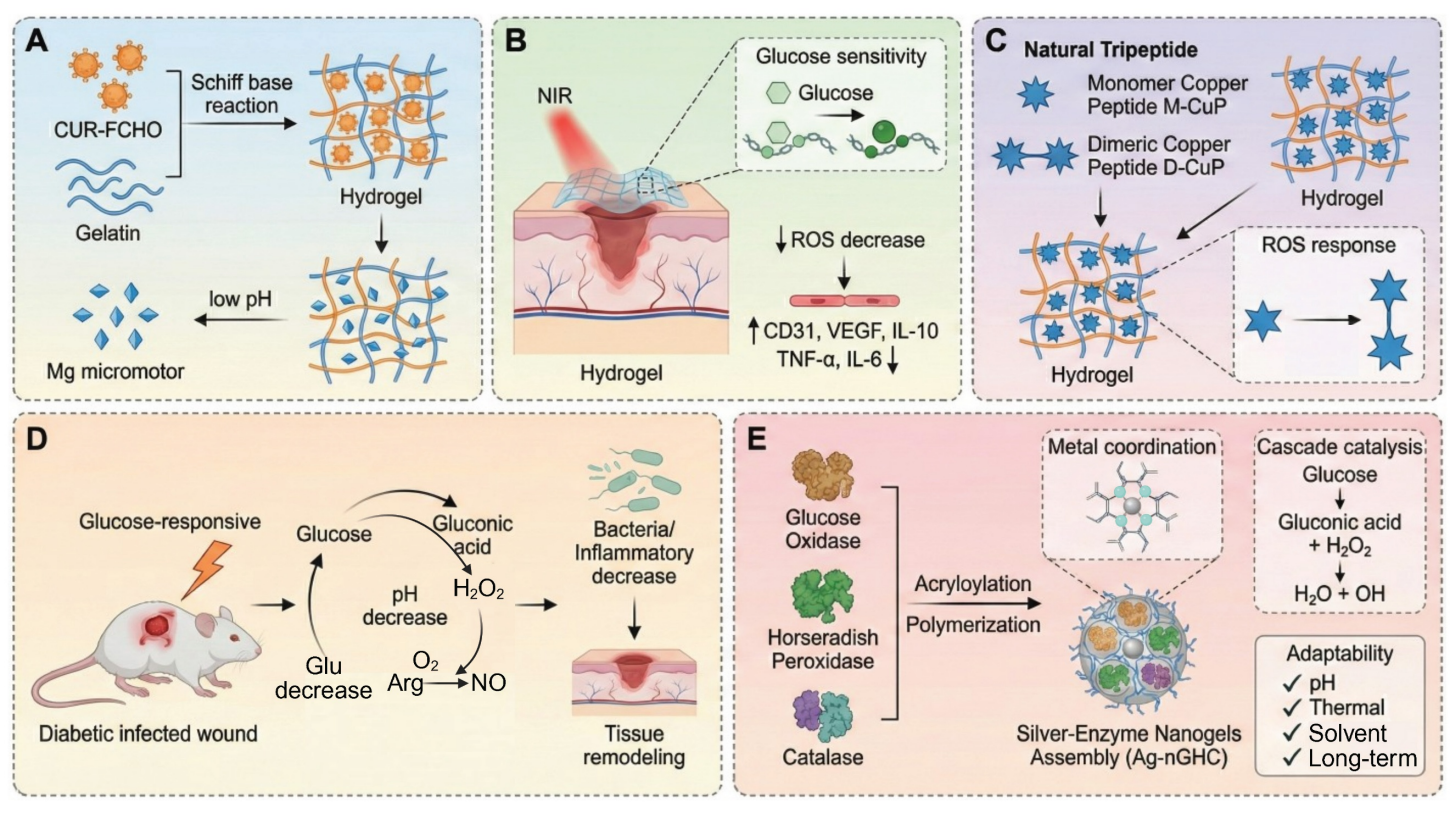
Classification based on the number of cascade reactions. (**A-C**) Single-layer cascade reaction system. (**A**) pH-responsive hydrogel enabling Mg micromotor disintegration and therapeutic release under acidic diabetic wound conditions. (B) The glucose-responsive antioxidant hydrogel for diabetic wound healing. HA-PBA provides glucose sensitivity, enabling dynamic binding with myricetin. (**C**) ROS-responsive hydrogel. (**D**) Dual cascade system of a dual-responsive AZG-Gel hydrogel that responds to hyperglycemia and high pH by GOx-mediated glucose oxidation. (**E**) Multilayer cascade reaction system of a multi-responsive Ag-nGHC nanogel system, responding to hyperglycemia, hypoxia, and bacterial infection via a cascade of GOX/HRP/CAT enzyme reactions. Created in BioRender. Jia, J. (2025) https://BioRender.com/f9fv3g2.

During the acute infection phase of diabetic wounds (within 48 hours), rapid acidification of the local microenvironment (pH=5.5 - 6.5) and the formation of biofilms by drug-resistant bacteria such as MRSA and Pseudomonas aeruginosa represent the main threats 179. In these scenarios, single stimulus responsive systems, which work through a single signal trigger mechanism, are often preferred because they are fast acting. Although these systems are not true cascade reactions, they are fast, targeted responses. For example, hyaluronic acid-based pH-responsive silver gel (HA-Ag) releases Ag^+^ ions via Schiff base bond cleavage in the acidic microenvironment with a peak concentration of 2.8 µg/mL in 24 hours and clears 99.3% of MRSA. However, residual Ag^+^ suppresses the migration of fibroblasts and retards the regeneration of the epidermis 180. Similarly, the Gel-FCHO/Mg-motor GCM hydrogel decomposes the Schiff bases at wound pH 5.5-6.5, while releasing curcumin-containing micelles and H₂ produced by Mg micromotors. Yet, rapid Mg corrosion threatens to cause an alkaline shift (pH > 8), which results in loss of peptide stability and requires buffering of microdomains to keep local pH (**Figure 6A**) 157. Another interesting design, a thermosensitive chitosan-tobramycin gel, induces a hydrophobic-hydrophilic transition at body temperature, which slowly releases tobramycin that penetrates biofilms with a 92% clearance rate and decreases local glucose concentration by 19% 181. The major benefits of these single-stimulus systems are their ease of synthesis and speed of response, which make them especially useful in the outpatient setting for acute treatment of superficial ulcers. However, their linear release kinetics can lead to sudden release of the drug, which could potentially cause local oxidative stress, and they cannot control the release of inflammatory factors, which limits their efficacy in chronic wounds. As a complementary strategy, the PEG-DA/HA-PBA/MY hydrogel takes advantage of the glucose-induced borate cleavage to release myricetin selectively under hyperglycemic conditions (≥15 mM) to reduce ROS without stopping physiological H_2_O_2_ bursts. Nevertheless, its release drops dramatically below 8mM glucose, requiring secondary triggers during normoglycemic phases (**Figure 6B**) 158. Meanwhile, the G/D-CuP hydrogel is based on dimeric CuP connected through ROS-cleavable bonds, where CuP is released only when H_2_O_2_ concentration is over 100 µM, hence coupling the anti-inflammatory and angiogenic effects. Dimerization provides increased protease resistance but can inhibit rapid diffusion in deep wound fissures (**Figure 6C**) 92. Other single-layer systems have been based on thermal or enzyme-mediated cascade activation, e.g. temperature-triggered release of antibiotics, which maintains ROS production and wound closure 181, 182. Compared to multilayer designs, these single-stimulus systems have simpler synthesis and quicker responses, and therefore are well-suited for early infection control. However, their linear release profiles and single level of activation limit their capacity to control inflammation and tissue regeneration over the long-term. Therefore, future research should be focused on the incorporation of secondary triggers or self-feedback mechanisms to increase the temporal adaptability and therapeutic precision of single-stimulus responsive systems.

#### 4.1.2 Dual cascade system

When the wound enters the subacute inflammatory phase, the vicious cycle of hyperglycemia, ROS overload, and hypoxia continues to be a critical limitation to wound healing. Dual cascade systems provide a major advance by allowing the modules of treatment to be activated in stages, which allows for energy self-supply and functional synergy. For example, the copper peroxide--GOx (CP@GOD) system uses GOx to catalyse glucose into H_2_O_2_, which is then decomposed by the CP nanozymes into hydroxyl radicals (•OH) and Cu^2+^ ions. The •OH result in a sterilization rate of over 99% against Staphylococcus aureus whereas Cu^2+^ ions trigger the HIF-1α pathway, which results in an 180% increase in the expression of the growth factor "VEGF" and, consequently, in the process of angiogenesis. In diabetic mouse models, this approach reduced the wound healing time to 14 days and greatly improved the vascular density 183. Another innovative design, the photodynamic ROS-responsive nanogel (Ce6@ZIF-8), takes advantage of the acidic microenvironment in the wound to release the photosensitizer Ce6. When activated by NIR light, Ce6 produces oxygen and can be used to target eradication of MRSA. At the same time, the photothermal effect promotes the orderly deposition of collagen, thereby reducing the formation of scars 184. The main benefit of these systems is the temporal control: the first infection control is performed by the antibacterial module, then the regeneration of factors to prevent interference with the therapeutic effect. However, synchronization of the kinetics of dual reactions is a technical challenge. For example, the injectable nanoreactor hydrogel Arg@Zn-MOF-GOx Gel (AZG-Gel) orchestrates two consecutive cascades: (1) GOx converts the hyperglycemic glucose into gluconic acid and H_2_O_2_, rapidly lowering local pH; (2) disassembly of Zn-MOF, triggered by the acid, releases arginine which consumes H_2_O_2_ to produce NO, thus ablating biofilms and inducing angiogenesis without the need for exogenous ROS. Although this all-in-one platform has a wound closure rate of 98.7% within 14 days, premature Zn-MOF degradation below pH 5.0 poses the risk of an NO burst that may induce transient cytotoxicity, underscoring the necessity of buffering microenvironments for clinical translation (**Figure 6D**) 159. Similarly, the rate of H_2_O_2_ generation in the CP@GOD system should be carefully balanced with the release of Cu^2+^ to avoid excessive free radical damage to surrounding tissues. Moreover, endogenous catalase activity is in competition during the decomposition of H_2_O_2_, which could decrease the efficiency of the cascade 183.

Dual-layer cascade hydrogels are made of different functional domains that act synergistically to spatially organize antibacterial, anti-inflammatory and regenerative effects. Compared to single layer systems, which provide fast response but mainly local action, these dual layer architectures can provide sequential therapeutic regulation and prolonged drug release. However, the fabrication of heterogeneous material layers of different swelling and degradation characteristics can cause interfacial instability and inconsistent cascade activation. Achieving synchronization of biochemical reactions within layers poses serious challenges in control, which raises processing complexity and hinders reproducibility. Despite promising therapeutic outcomes shown in preclinical studies, dual-layer cascade systems still suffer from challenges in structural optimization and scalable manufacturing. Therefore, future studies should aim to increase interfacial bonding, coordinate permeability between layers, and standardize production processes to increase stability and promote clinical translation.

#### 4.1.3 Multilayer cascade reaction system

The multiple cascade reaction system is a biomimetic catalytic network, which incorporates multiple enzymes or enzyme-like catalysts (e.g., nanozymes) in confined domains to build a multi-step, self-sufficient biochemical reaction chain for synchronous regulation of the diabetic wound microenvironment. Its main characteristics are: (1) multifunctional coupling, realized by synergistic enzyme catalysis (e.g., GOx/hydrogen peroxidase), nanomaterial catalysis (e.g., Pt/CeO_2_), and controlled release of bioactive molecules, which can achieve time-sequenced intervention, including glucose reduction, antioxidation, anti-inflammation, and tissue regeneration; (2) pathological responsiveness, induced by disease-specific signals such as hyperglycemia and ROS to achieve targeted metabolic regulation.

Given the multidimensional pathological characteristics of diabetic wounds such as hyperglycemia, inflammation, infection, and impaired angiogenesis, therapeutic systems need to have dynamic and synergistic intervention capabilities depending on wound stages. Single-layer cascade systems (e.g., glucose-activated antibacterial gels) consume glucose quickly and release Ag^+^ through enzymatic reactions, killing 99% of MRSA within 48 hours, but are not multifunctional enough for later stages of repair 112. Dual cascade systems (e.g., pH/ROS-responsive hydrogels) that allow synergistic ROS clearance and sustained exosome release with temporal control to facilitate orderly anti-inflammatory and pro-angiogenic responses with a 40% increase in healing rate in animal models. Multi-layer cascade systems combine glucose response, antioxidation and epithelial regeneration, reducing wound closure to 8 days in diabetic mouse models, a good example of whole chain management 168. The Ag-nGHC platform demonstrates a multilayer cascade with stage-adaptive responsiveness (**Figure 6E**) 160. Its triple-enzyme nanogel structure allows for sequential glucose depletion, oxidative sterilization and oxygen regeneration, matching therapeutic activities to the wound healing process timeline, from infection control to tissue regeneration. Importantly, the high biocompatibility of the system stems from the incorporation of native enzymes, without the immunogenicity that is common with inorganic nanozymes. However, the inherent fragility of native proteins requires encapsulation strategies for *in vivo* sustained activity. This approach offers a new blueprint for enzyme-based therapeutics in diabetic wound care with potential applications beyond diabetic foot ulcers in burns and ischemic ulcers.

Hydrogel cascades multi-layer cascade hydrogels have emerged as a new advancement in regenerative medicine, in which the multiple layers with functional specifications permit hierarchical coordination of therapy because of spatially and temporally discrete responses 168. These hydrogels are more functionally complex and allow sequential delivery of various biochemical signals than dual-layer systems. The increased structural hierarchy however causes problems including kinetic imbalances, diffusion mismatch across layer and buildup of intermediate byproducts all of which may cause incomplete or slow cascade activation 165. Also, mechanical discrepancies and fabrication issues do not allow reproducibility and stability.

Moreover, excessive combination of various stimuli, catalytic modules and signaling pathways is dangerous to biological incoherence. The responsive units can interfere with each other, leading to kinetic interference or overlapping signals, which can disrupt the redox balance or result in immune overactivation. These unintended interactions may backfire on the therapeutic benefits and may augment the biosafety issues. Although multi-layer hydrogels are promising as programmable wound therapies, inter-module interference and the complexity of the system have inhibited their clinical translation. Thus, the functional simplification, decoupling of reaction pathways, and rational coordination of response modules should be made in the future to maintain controllability and biological harmony.

### 4.2 Classification based on cascade reaction mechanism

The pathological complexity of diabetic wounds demands systems of treatment capable of dynamically and multidimensionally controlling the underlying mechanisms 25, 105. Depending on the principle of their triggering, the cascade reaction mechanism can be classified into three groups namely natural enzyme driven, biomimetic catalytic, and physicochemical response. Biocatalysis, material biomimetics and non-enzymatic cascade processes are precise interventions. The concerted integration of these mechanisms not only is commensurate to the pathological features stage-specific for the wound, but also overcomes the inherent limitations of single-modality treatments.

#### 4.2.1 Enzymatic cascade reaction: precise activation of endogenous substrates in wounds

**Figure 7:**
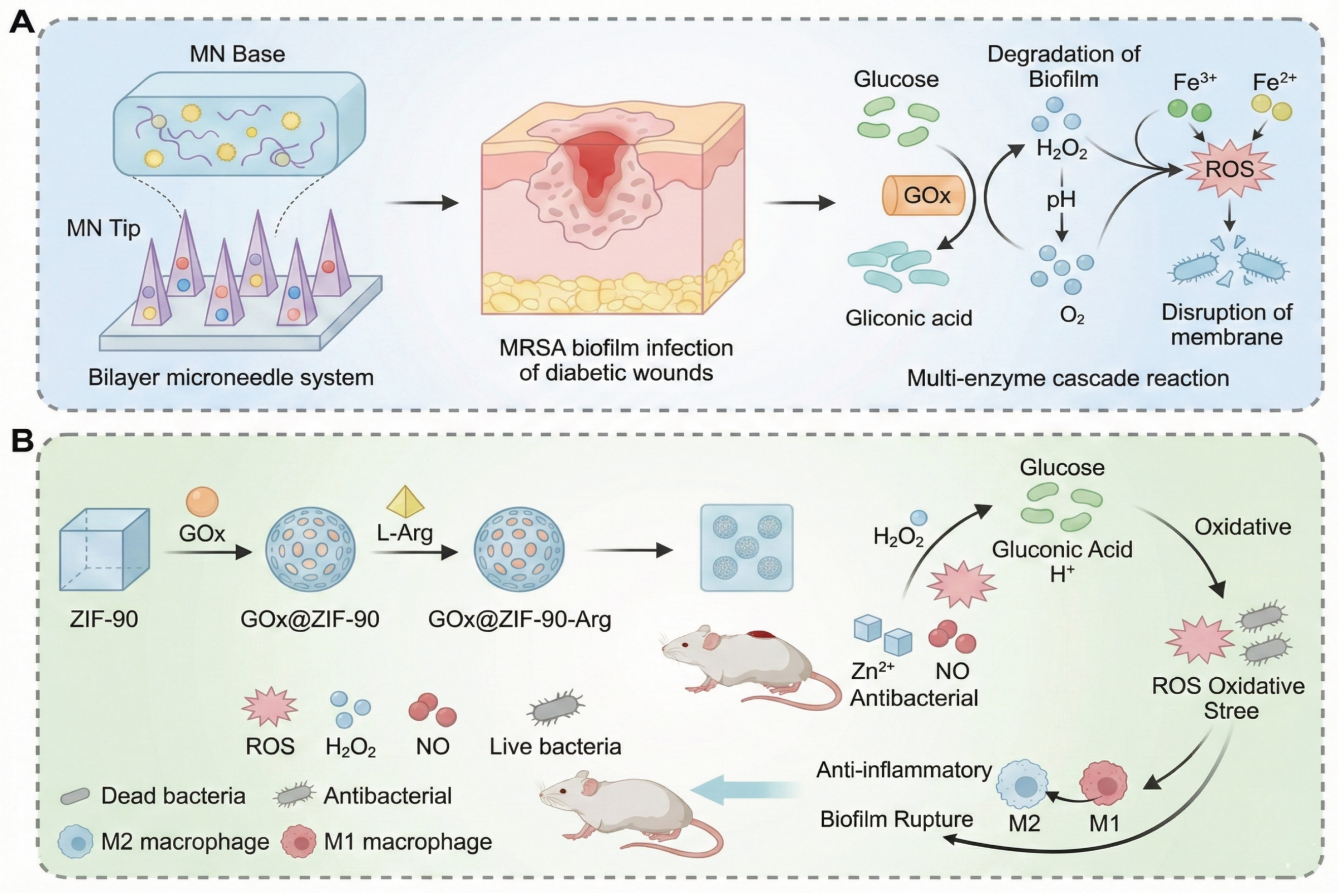
Enzymatic cascade reaction: precise activation of endogenous substrates in wounds. (**A**) Schematic illustration of the multi-enzyme cascade microneedle system for chronic wound treatment. (**B**) Schematic representation of the enzyme cascade reaction-based hydrogel (Hyd-GZA) for treating diabetic wounds infected with bacterial biofilms. Created in BioRender. Jia, J. (2025) https://BioRender.com/iizi4ou.

The cascade system based on natural enzymes has shown significant biocompatibility in acute phase applications. For example, the dual enzyme system of GOx and HRP converts high-concentration glucose into antibacterial reactive oxygen (·OH) through two-step catalysis, reducing the bacterial load on the wound surface by 98% in a diabetic mouse model (only 58% in the control group), while promoting collagen deposition to accelerate healing 186. In this process, GOD first catalyzes glucose to produce H₂O₂, and then HRP decomposes it into ·OH with a broad-spectrum bactericidal effect, which not only achieves the goal of anti-infection, but also alleviates high glucose toxicity by reducing local glucose. Complementing the above GOD-HRP platform, an α-amylase/GOx-coupled chemodynamic patch has been engineered to sequentially degrade EPS polysaccharides and hyperglycaemic glucose. α-amylase first liquefies the biofilm matrix, liberating glucose that is immediately oxidised by GOx-immobilised MOF nanoneedles to generate local H₂O₂; subsequent Fenton-like chemistry converts H₂O₂ into cytotoxic •OH without exogenous H₂O₂, achieving 99.9 % MRSA eradication while lowering ambient glucose by 45 %. Nevertheless, the burst release of •OH at pH 5.5-6.0 transiently elevates oxidative stress, demanding ROS-quenching back-up layers to protect nascent granulation tissue (**Figure 7A**) 185. Alternatively, a pH-driven NO-releasing cascade was recently constructed by co-encapsulating GOx and l-arginine within acid-labile ZIF-90 (GOx@ZIF-90-Arg) 158. GOx-mediated glucose oxidation simultaneously consumes glucose and acidifies the microenvironment, triggering ZIF-90 disintegration to release Zn²⁺ and H₂O₂; the latter drives arginine oxidation to produce NO, which disperses 96% of pre-formed biofilms and enhances angiogenesis via cGMP signalling. The system attains 98.7% wound closure within 14 days, yet the rapid ZIF dissolution below pH 5.0 risks premature payload exhaustion, necessitating pH-buffering hydrogels or core-shell architectures to prolong therapeutic windows (**Figure 7B**) 174. Enzymatic cascade reactions are considered to be one of the most biologically compatible strategies for wound regulation. These reactions use endogenous substrates to trigger sequential biochemical reactions with high spatial selectivity. In the setting of diabetic wounds, multi-enzyme systems, such as GOx/peroxidase or superoxide dismutase (SOD)/catalase cascades, have been shown to convert excessive glucose or ROS into controlled signals 158. This process allows the modulation of oxidative stress, oxygen supply, and inflammation *in situ*. The efficacy of these designs in promoting self-sustaining biochemical feedback has been demonstrated, especially in promoting angiogenesis and tissue regeneration under conditions of hyperglycemia and hypoxia. However, the use of enzymatic cascade hydrogels is frequently limited by their complex kinetic processes and sensitivity to environmental variations. The activity and stability of natural enzymes can be affected by variation of pH, changes in temperature and by proteolytic degradation, resulting in unpredictable catalytic efficiency *in vivo*. Moreover, the over-integrated cascade networks with multiple catalytic modules can be affected by issues like kinetic crosstalk, substrate competition or imbalanced reaction rate. These issues may result in a reduction of controllability and potentially the production of unwanted intermediates. These effects can lead to local redox homeostasis or oxidative and immune stress which can reverse the desired therapeutic effects.

Multi-enzyme reactions are more specific to substrates compared to single-enzyme or photo/ROS-driven cascade systems, but they require strict stoichiometric control and local cofactor balance, which is a challenge to their standardization in clinical application. To address these issues, subsequent research should aim at stabilizing enzymes, either through biomimetic confinement or nanozymatic replacement, and rational pathway decoupling to guarantee catalytic precision and biological harmony in general.

#### 4.2.2 Enzyme-mimetic cascade reaction: an engineering revolution in biomimetic catalysis

**Figure 8:**
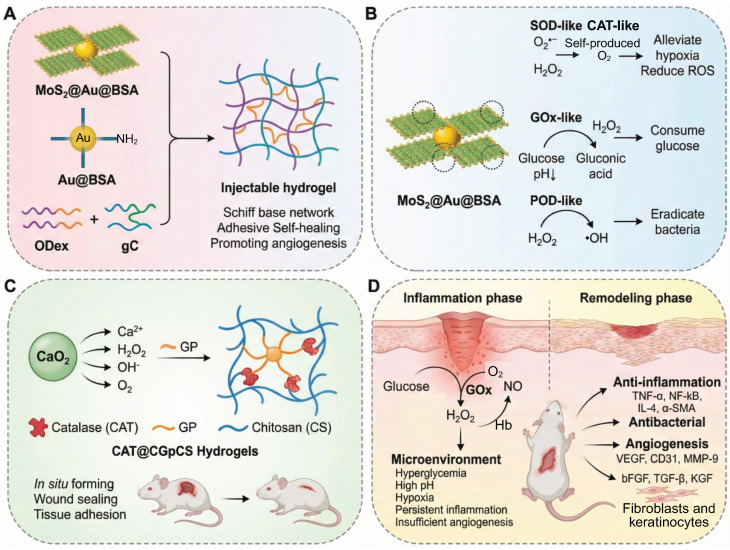
Enzyme-mimetic cascade reaction: an engineering revolution in biomimetic catalysis. (**A-B**) Schematic representation of the enzyme-mimicking cascade reaction: Au nanoparticles mimic glucose oxidase to catalyze glucose into gluconic acid and H_2_O_2_, while MoS_2_@Au@BSA mimics peroxidase to convert H_2_O_2_ into •OH for bacterial eradication. (**C**) Schematic illustration of the enzyme-mimicking cascade reaction: CaO₂ mimics catalase to convert H₂O₂ into O₂, enhancing oxygen supply for rapid hemostasis and wound healing. (**D**) Schematic diagram of the enzyme-mimicking cascade reaction: Hb mimics peroxidase to catalyze H₂O₂ into NO, while GOx converts glucose into H₂O₂, enabling glucose-activated NO release and microenvironment regulation. Created in BioRender. Jia, J. (2025) https://BioRender.com/ofjnlrk.

To overcome the instability and limited catalytic turnover of natural enzymes, biomimetic cascade systems employ engineered materials to reproduce and enhance enzymatic redox activity through spatial confinement and controlled electron transfer. MoS₂@Au@BSA nanozyme-hydrogel conjugates integrate GOx-, POD-, CAT- and SOD-like activities in one scaffold, consuming glucose, releasing •OH and O₂ while adapting to pH shifts; ultrasmall Au domains boost catalysis but the multi-step synthesis elevates cost and batch variability (**Figure 8A-B**) 188. The ultrafine Au domains act as electron relays between Mo and S centers, accelerating charge transfer and enhancing peroxidase-like activity. The injectable genipin-chitosan hydrogel is cross-linked and activated by CaO₂ *in situ*, establishing a self-sustaining O_2_/Ca^2+^ release network. The confined gel matrix with nanoscale channels localizes oxygen and facilitates ion diffusion, enhancing gelation kinetics and mechanical integrity while accelerating coagulation; the resulting hyperoxia and Ca²⁺ further promote rapid hemostasis and macrophage M2-driven angiogenesis. However, optimization of CaO_2_ dosage and control of alkaline by-products are still required to balance the catalytic efficiency and biocompatibility (**Figure 8C**) 189. The iron single-atom nanozyme (Fe-SAzyme) gel is a remarkable advance in the world of catalyst speed. Within the acid microenvironment of diabetic wounds (pH 5.5-6.5), it has been found to mimic the activity of CAT, thus degrading H_2_O_2_ to O_2_, and reversing hypoxia. Additionally, at a neutral pH, it has POD mode, which results in the generation of ROS. The Fe-SAzyme shows a quantitative tendency of H_2_O_2_ decomposition with a turnover frequency of about 1.8 x 10^4^ s^-1^. This is almost four times the rate of conventional Fe_3_O_4_ nanozymes under similar conditions. As a result, it has an MRSA inhibition rate of 99.99%, indicating its remarkable efficiency in antibacterial applications 191. The metastable nanocube-based hydrogel makes use of a biofilm environment-responsive cascade. In this cascade, Cu-Fe dual sites catalyze Fenton-like reactions to produce the formation of the reactive oxygen species (ROS) •OH that degrade DNA and disrupt biofilm structures. Electron redistribution between copper (Cu) and iron (Fe) centers creates a redox-relay network for speeding up the Fenton reaction rate constant to about 1.2 × 10^6^ M^-1^ s^-1^, which is about 2.8 times faster than that of Fe^2+^ alone 192. Meanwhile, the mild hydroxyl flux at the biofilm periphery reprograms macrophages toward a pro-inflammatory phenotype. This reprogramming eliminates residual bacteria and achieves a complete clearance of implant infections. Recent advances have also focused on constructing cascade catalytic hydrogels that regulate the diabetic wound microenvironment through glucose-triggered NO generation. For instance, a GOx/hemoglobin artificial multienzyme nanoflower integrated CMCS/OSA hydrogel (COH-GB) converts excess glucose into gluconic acid and H₂O₂, and subsequently drives H₂O₂-mediated NO release from hydroxyurea under hemoglobin catalysis, achieving synchronized glucose consumption, ROS scavenging, antibacterial activity, and angiogenesis promotion in MRSA-infected diabetic wounds.

The hierarchical nanoflower structure enhances enzyme stability and cascade efficiency, providing a model for multienzyme confinement strategies in biomimetic catalysis (**Figure 8D**) 190. Recent studies employ MOFs as confined catalytic scaffolds that enhance active-site accessibility and facilitate charge transfer via spatial confinement and defect modulation. For instance, Co₃O₄ nanozymes confined within MOF channels have been shown to enhance the utilization rate of H₂O₂ from 21% to 68%, thereby achieving a 3.2-fold improvement in catalytic efficiency through defect-induced electronic redistribution 193.

A comparison between enzyme-mimetic systems and natural enzyme cascades shows better mechanical robustness, long-term catalytic stability and denaturation resistance in the former. However, an expansion in substrate range often leads to a diminution in substrate specificity and fine kinetic coordination between reaction modules. Despite the improvements mentioned above that are measurable in quantifiable terms, the majority of biomimetic cascade based on hydrogel lacks any standard kinetic parameters that can be used to measure cascade efficiency. The turnover rate, ratios of catalytic constants (k_cat_/k_m_), reaction half-life (t_1/2_) and synchronization coefficients are rarely reported, which makes it challenging to compare systems across and to rationally optimize them. The present gap in the knowledge can be sealed by the future research that will involve the quantitative modeling of the electron transfer kinetics together with the rational design of the defects and spatial confinement. This combined methodology will guarantee the catalytic specificity without leakage of electrons or cross reaction. At the same time, the creation of bio-degradable or self-limiting nanozymes, as well as the creation of integrated evaluation standards, will be essential to sustaining biological coordination and advancing to clinically viable, safe and intelligently controlled biomimetic therapies.

#### 4.2.3 Chemical/physical trigger cascades: non-enzyme-dependent responses

Compared to biocatalysis-based systems, physicochemically triggered cascades have unique benefits in complicated surroundings by exploiting non-enzymatic mechanisms. For example, a pH/temperature dual-responsive PVA hydrogel takes advantage of the natural microenvironmental fluctuations of the wound: the tertiary amine groups are protonated in the acidic exudate (pH 5.5), allowing for the rapid release of 90% of chlorhexidine within 6 hours to control infection. When the temperature reaches 37 °C, the lysozyme is activated for the cleavage of pathogen peptidoglycan, which increases biofilm penetration 194. This design ingeniously integrates the antibacterial and anti-biofilm effects by synergistic environmental cues. A more sophisticated light controlled, three-level system, provides temporally precise intervention through multi-wavelength illumination. NIR (808 nm) light causes the photothermal phase transition of the gold nanorods, releasing NO, which causes vasodilation and enhances blood flow by 40% 195. The concomitant temperature increase causes secondary cross-linking of the hydrogel that allows slow release of insulin-like growth factor 1 to promote tissue regeneration. Subsequently, 650 nm blue light is used to excite conjugated polymers to produce reactive nitrogen species that effectively kill residual biofilms. Preclinical studies indicated this approach shortened wound closure time to 14 days without causing the bacterial imbalances that are commonly associated with traditional antibiotics. However, clinical translation is still limited by the device dependency: accurate light control necessitates specialized laser sources, and photothermal material efficiency is reduced after repeated cycles of use 196, 197.

In summary, these three types of cascade reaction-based systems have different characteristics: enzymatic cascade systems use the endogenous biomolecules to achieve highly specific reactions, which are suitable for early acute wound intervention; quasi-enzymatic systems use biomimetic catalysts to provide stable and controllable reaction pathways, which are suitable for continuous treatment of chronic wounds; chemical/physical trigger systems use multiple stimuli to comprehensively regulate the reactions from antibacterial and anti-inflammatory effects to tissue remodeling. Future developments should focus on optimizing the coordination of the timing and precision of responses across cascade steps, optimizing the long-term biocompatibility and safety of materials and incorporating embedded sensors with artificial intelligence (AI) to allow real-time monitoring of wound dynamics. Such advancements will lead to a more accurate and efficient multidimensional intervention platform in the care of diabetic wounds. This multi-level, intelligent cascade reaction strategy shows great promise for the further development of the widespread clinical application of responsive hydrogels, which will ultimately bring significant therapeutic benefits and improve the quality of life of patients.

### 4.3 Classification based on therapeutic phase targeting: chronological cascade response and synergistic cascade response

**Figure 9:**
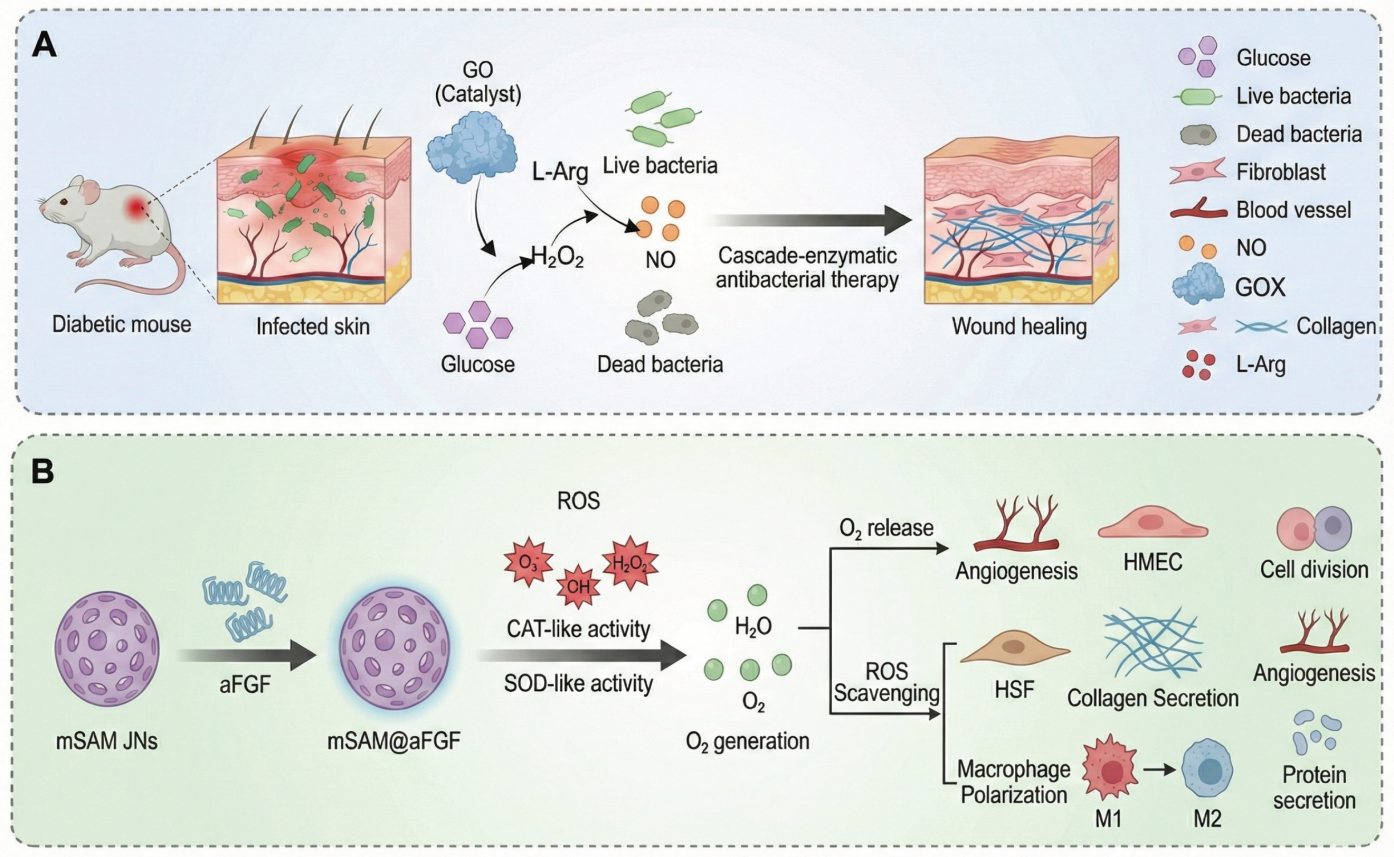
Classification based on therapeutic phase targeting: (**A**) Chronological cascade response of the time-sequential cascade reaction in the GOA@HG cryogel system: Glucose oxidase (GO) first catalyzes glucose to produce H₂O₂, which subsequently reacts with L-Arginine (L-Arg) to generate nitric oxide (NO). (**B**) Synergistic cascades mechanisms of the mSAM@aFGF hydrogel system in promoting diabetic wound healing. Created in BioRender. Jia, J. (2025) https://BioRender.com/pc6iij5,

The process of chronic diabetic wound healing is dynamic, staged and proceeds through initial infection and sustained inflammation to chronic regenerative impairment. As a consequence, it has become important to develop treatment systems which can react to particular stages to be able to target and intervene in the chronic wounds of the diabetic foot 198. Intelligent systems with temporal responsiveness and synergistic mechanism coupling have become a promising approach, and is known as cascade therapy, enabling dual-dimensional accuracy in time and space. This strategy has contributed to a great enhancement in the effectiveness and safety of clinical interventions. An example of such a system is the glucose/pH dual-responsive hydrogel system (PBA-PCL/Chitosan) which enables drug release on a stage-by-stage basis (**Figure 9A**). It preferentially responds to high levels of glucose in infection by releasing metformin nanoparticles to inhibit the formation of AGEs, reducing oxidative stress before moving into tissue repair phases 199. Another interesting system is a multifunctional nanozyme hydrogel based on MnO_2_-Au-mSiO_2_ Janus nanoparticles encapsulated with acidic fibroblast growth factor (aFGF), which synergistically neutralizes ROS, releases oxygen, and promotes angiogenesis, macrophage modulation, and fibroblast proliferation, providing both antimicrobial and pro-regenerative actions for diabetic wound therapy at the same time (**Figure 9B**) 200.

#### 4.3.1 Chronological cascade response: dynamic adaptation of pathological processes

The pathological course of diabetic wounds is generally acidification and infection, oxidative stress explosion, and poor regeneration, which correspond to the characteristic stages of diabetic wounds, such as the early stage of acidic microenvironment, the middle stage of high ROS, and the late stage of the lack of angiogenesis. Therefore, the design of sequential cascade systems demands high sensitivity to identify precisely the markers of wound progression, such as pH and enzyme activity, and selectively to switch on the functional modules according to the therapeutic sequence of "anti-infection → anti-inflammation → regeneration promotion," to avoid cross-stage interference.

A representative example is the temperature-directed spatiotemporal cascade system, which makes use of the synergistic responses of a PNIPAM thermosensitive layer and drug loaded nanofibers 27. In the acute phase, the thermosensitive layer quickly shrinks and releases ciprofloxacin to inhibit deep infections. During the repair phase at 37 °C, nanofibers degrade to release EGCG which scavenges ROS and stimulates fibroblast migration. Preclinical studies showed that this system is highly effective in reducing the recurrence of infection in diabetic rabbit models.

Another interesting example is the enzyme-triggered bioorthogonal system, which uses the β-lactamase secreted by pathogens to activate a two-stage drug release 201. First, the cleavage of the β-lactam bond produces ceftazidime acting against Gram negative bacteria. Thereafter, a catechol intermediate is formed, causing acidification of the microenvironment to pH 5.2, leading to controlled release of salicylic acid, which is effective in penetrating and eradicating resistant bacterial biofilms to depths of up to 200 µm. This approach resulted in an 84% biofilm removal rate and a significant reduction of systemic antibiotic use.

Sequential cascade systems provide smooth switching between modules based on the correct detection of microenvironmental changes, such as pH and enzyme concentrations, which prevents the functional overlap and the adverse side effects 87, 113. Although environmental marker-based stage switching minimises mutual interference between therapeutic modules, there are still translation issues: multi-stage material engineering raises concerns about cost, scale and reproducibility. The next generation should be simplified and adaptive architectures with the ability to provide temporal feedback in order that the materials can automatically adapt to the natural healing rhythm of the wound yet provide biological coordination.

#### 4.3.2 Synergistic cascades: mechanism coupling in the spatial dimension

In order to address the above challenges, synergistic cascade systems are used to combine spatial coupling and functional complementarity strategies to realize multi-target and complementary interventions on the time and space dimensions. The most common one is the gas-synergistic hydrogel system, which relies on copper peroxide nanozymes (CuO_2_) to decompose endogenous H_2_O_2_ to generate •OH and oxygen, and further catalyze nitrate to generate NO 202. The direct effect of the •OH radicals is to disrupt the bacterial membranes, which inhibits the growth of the MRSA (inhibition zone of 18 mm), whereas NO induces the vasodilation, increasing the velocity of the blood flow by three times and suppressing the inflammatory action, reducing IL-6 levels by 72%. In animal models, this system has been demonstrated to have 95% wound closure rate in 28 days in diabetic animals and 2.3-fold increase in blood vessel density of CD31^+^.

Although synergistic cascade systems have a huge potential in the simultaneous multi-target control of diseases, they also face new challenges, which are caused by the rise of structural and functional complexity. Redox, gas, and immune pathways crosstalk may lead to non-linear feedback loops that may cause oscillatory or competing biological signals, which can impair therapeutic efficacy or safety. Moreover, the energy and co-ordination requirements required to attain multi-modal activation may exceed physiological limits, raising concerns on long-term biosafety. The way forward in research is in the development of minimalist, but collaborative architectures that can be multifunctional by means of intrinsic material coupling rather than overly stacked modularly. In addition, the quantitative models to align the cascade kinetics with the physiological schedule of the wound are essential. Such design principles will enable a shift in the empirical assembly to the real intelligent integration in wound therapeutics.

## 5. Critical Challenges and Future Outlook

Despite great efforts on the development of microenvironment-responsive hydrogels for diabetic wound therapy, their clinical translation is at an early stage. While many of these systems show encouraging efficacy in rodent models, few have been advanced beyond preclinical validation. Bridging the gap between success in the laboratory and in the clinic involves solving a number of important challenges, including the translational divide, scalability of manufacture processes, developing standardized evaluation models, and finding the right balance between multifunctionality and biocompatibility.

### 5.1 The “Valley of Death” between preclinical success and clinical application

Although there have been many studies showing accelerated wound closure, enhanced angiogenesis, and modulated immune responses in small-animal models, few have been translated to clinical trials. This gap commonly known as the “valley of death” can be attributed to some underlying differences between the controlled conditions of laboratory experiments and the heterogeneous and multifactorial nature of chronic human wounds. The ulcers of diabetics in patients are typically typified by fluctuating infection patterns, intricate exudate formulations and intricate immune reactions which cannot be completely reproduced in rodent models. In addition, numerous high-technology hydrogels include other substances like enzymes, MOFs or nanozymes, the long-term metabolic behavior and immunogenicity of which are not known. The lack of complete toxicological information and a lack of uniformity in reporting biodegradation pathways further complicate regulatory approval. To address this translational gap, it is necessary not only to understand the mechanisms that are involved in a better way, but also to collaborate on an interdisciplinary level between materials scientists, clinicians and regulatory experts to come up with hydrogels that are compatible with clinical manufacturing and safety assessment standards. It is worth noting that recent preclinical trials seldom use comorbidity models that are more representative of clinical conditions of diabetic patients (concurrent cardiovascular disease, obesity, immune dysregulation or peripheral neuropathy). The lack of these complex systemic conditions is a major limitation to the predictive power of current models and a major hurdle in bridging the translational "valley of death."

### 5.2 Manufacturing and scalability: cost and complexity barriers

The second big problem is the cost and manufacturability of such highly engineered systems. Multi-responsive and cascade-reaction hydrogels often require complex synthetic methods, multiple purification processes and addition of costly catalysts, enzymes or nanoparticles. These contribute to the cost of production much higher than in the case of conventional wound dressings and are a considerable obstacle to scalability in the circumstances of good manufacturing practice (GMP). Also, the batch-to-batch difference of natural polymers or biologically-derived components contributes to the problems of reproducibility and quality control. The way forward must be based on more direct and simplified methods of fabrication, including click chemistry, microfluidic fabrication and 3D bioprinting where composition and spatial patterning can be precisely controlled. The advent of printable hydrogel inks and self-healing dynamic crosslinking chemistries promises to provide the ability to scale up production with less variability. Coupling these process innovations with automated quality control and continuous manufacturing systems will be critical for successfully taking these advanced materials from the laboratory to clinical application.

### 5.3 Need for standardized large-animal model testing

Preclinical testing of diabetic wound dressings is still largely based on streptozotocin-induced rodent models which do not adequately recapitulate the complex pathophysiology of human chronic ulcers. Significant anatomical and physiological differences, including skin thickness, vascular density and immune cell composition, between rodent and human tissue often result in overestimated healing rates in these models. To better close this translational gap, standardized large animal models, such as diabetic minipigs and canines, are urgently needed. These models better resemble the size, biomechanics and inflammatory responses of human wounds and, therefore, provide more reliable predictions of clinical efficacy and safety. In addition, more meaningful cross-study comparisons will be made possible through the development of coherent evaluation criteria, which will include quantitative criteria, such as wound closure kinetics, neovascularization indices, and inflammatory cytokine profiling. The collaboration between countries and compliance with the regulatory standards, i. e., ISO 10993 on biological testing and NIH translational studies, are mandatory to align the preclinical testing and fast track regulatory approval. In addition to conventional wound closure outcome measures, functional outcomes (e.g. nerve regeneration, loss of tactile sensation, long-term scar quality and biomechanical strength) should be also measured in large animal studies, which are relevant to the long-term clinical outcome of diabetic wound therapies.

### 5.4 Balancing multifunctionality and biocompatibility

With the development of hydrogel designs towards multifunctional and cascade-based systems, the significance of a delicate balance between enhanced functionality and biocompatibility gains significance. Although photothermal agents, transition metal nanozymes, or conductive fillers can significantly improve antibacterial, antioxidant and pro-angiogenic properties, these components can also lead to cytotoxicity or chronic inflammatory reactions. Problems like overproduction of ROS, retention of residual metal ions and uncontrolled byproducts of degradation pose a threat to the tissues around. To achieve a perfect balance between therapeutic efficacy and biosafety, therefore, it is necessary to select materials rationally and control release kinetics. The use of biodegradable enzyme mimics, natural polysaccharides and bioinspired dynamic covalent bonds are some of the possible approaches to reduce the toxicity and maintain responsiveness. Moreover, adaptive degradation profiles coupled with "on-demand" activation mechanisms guarantee that hydrogels perform their therapeutic functions effectively without long-term accumulation and disruption to the immune system.

### 5.5 Outlook: toward intelligent and personalized wound management

Future generations of responsive hydrogels can be expected to develop into intelligent, self-regulating therapeutic systems. The integration of AI, specifically predictive modeling, allows large datasets of the material compositions, reaction kinetics, and biological outcomes to be analyzed. This makes it easy to determine the best cascade parameters, including enzyme ratios, diffusion coefficients, and activation thresholds. By using machine learning algorithms in combination with extensive experimental databases, researchers can predict cascade dynamics and screen formulation candidates in an efficient way, substantially lowering the need for labor-intensive *in vivo* studies. However, the current application of AI-assisted modeling has been hampered by the lack of standardised and high-quality datasets as heterogeneity in experimental designs, data annotation and reporting format is a huge challenge.

Simultaneously, OoC platforms that can mimic the microenvironment of the diabetic wound, with its dynamic fluctuations of glucose, pH, and ROS, provide real-time evaluation of hydrogel functionality under physiologically relevant conditions. These microphysiological systems bridge the gap between traditional *in vitro* assays and animal models and offer a dynamic, human-relevant interface to monitor drug release, angiogenesis and immune responses *in situ*. Despite their promise, building multi-tissue OoC models that faithfully recapitulate the chronic wound milieu remains technically difficult, particularly in terms of keeping vascular, immune, neural, and epithelial components in concert.

The integration of the models driven by AI and the assessment of the models by ooC promises to speed up the transfer of complex cascade hydrogel systems between the conceptual phase and the clinic. Also, biosensing modules incorporated in hydrogels will allow feedback-based control to provide autonomous drug delivery and control the microenvironment. Hydrogel designs customized on patient-specific omics information, microbial flora profiling and wound metabolic signatures offer further refinement in accordance with individual healing patterns. With these approaches coupled with the development of three-dimensional bioprinting and microfluidic systems, heterogeneous and gradient-structured hydrogels will be produced that are more akin to the native tissue architecture.

Finally, the success of this undertaking in the long run is the establishment of methodologies of production that will be regulatory-compliant, cost-effective, and environmentally sustainable. In order to effectively cross the valley of death between preclinical innovation and clinical implementation, multidisciplinary cooperation between materials scientists, bioengineers, clinicians and policy makers is necessary. It is only through this type of combined approach that microenvironment-responsive hydrogels can leave behind the experimental prototype stage to become standardized, intelligent wound care products that can transform chronic diabetic wound care.

## 6. Conclusion

Although current studies show the superior performance of responsive hydrogels in controlled experimental conditions, the environment of diabetic wounds is highly heterogeneous and dynamic. These wounds have varying pH, oxygen gradients, enzyme activities and distributions of immune cells, all of which can have a significant effect on hydrogel responsiveness, diffusion, and degradation behavior. Such complexities frequently cause discrepancies in *in vitro* and *in vivo* outcomes. Therefore, future research should include more bio-relevant wound models, such as microfluidic chips, OoC systems, or large-animal chronic wound models, to better mimic the spatiotemporal heterogeneity of real wounds and to assess the adaptive responses of materials in physiologically realistic settings. Microenvironment-responsive hydrogels have become a promising approach for the treatment of chronic diabetic wounds. By sensing pathological cues, such as glucose, pH, ROS, and enzyme activity, these intelligent materials allow for spatiotemporally controlled drug delivery and the coordinated modulation of inflammation, angiogenesis, and oxidative stress. Over the past few years, progress in dynamic covalent chemistry, enzyme-mimetic catalysis and cascade reaction design has led to significant improvement in the therapeutic precision and performance of hydrogel dressings. Particularly, multi-responsive and cascade-reactive systems have started to mimic the complex feedback loops of the wound microenvironment, with the potential of comprehensive and adaptive repair across the healing process.

Nevertheless, there are still important challenges to overcome on the way to clinical translation, as discussed in Section 6. Bridging the translational gap, scalable and cost-effective manufacturing, large-animal testing standardization, and balancing multifunctionality and biocompatibility are important prerequisites for real-world application. Future progress will require interdisciplinary collaboration between materials science, biomedical engineering and clinical medicine to bring conceptual prototypes to the clinic. The combination of biosensing technologies, AI-driven modeling, and OoC evaluation has the potential to transform responsive hydrogels into personalized and self-regulating therapeutic platforms.

In summary, the development of microenvironment-responsive hydrogels represents a paradigm shift from the passive wound covering to a more dynamic, intelligent and precision-guided therapy. With further innovation and stringent translational validation, these materials are expected to become the next generation of bioactive dressings that will significantly enhance the management of diabetic wounds.

## Figures and Tables

**Table 1:** Representative single-stimulus microenvironment-responsive hydrogels for diabetic wound healing and their key physical properties (2024-2025). All values represent typical ranges reported in the cited literature, and may vary substantially depending on formulation composition and testing conditions.

Hydrogel type	Representative system	Core mechanism	Compressive strength (kPa)	Adhesive strength (N cm⁻²)	Swelling Ratio (%)	Degradation rate (% mass loss, days)	Ref. (Year)
Glucose-responsive	Zn-MOF-GOx cascade nanoreactor	GOx-catalyzed glucose oxidation triggers NO generation and microenvironment modulation	65 ± 5	1.8 ± 0.2	370 ± 25	45 ± 3 (7 d)	101 (2024)
Glucose-responsive	Multienzyme “nanoflower” hydrogel (GOx-activated NO)	Glucose-triggered NO release from multienzyme composite	72 ± 6	2.0 ± 0.3	380 ± 20	50 ± 4 (7 d)	109 (2025)
pH-responsive	TA/QCMC/OSA injectable hydrogel	Acid-base reversible dynamic bonds enabling pH-triggered release	60 ± 4	1.6 ± 0.2	350 ± 15	40 ± 5 (5 d)	102 (2024)
pH-responsive	Acid-responsive CST@NPs system	Acidic wound milieu triggers drug release from acid-labile construct	45 ± 3	1.2 ± 0.2	390 ± 30	42 ± 6 (7 d)	116 (2024)
Enzyme-responsive	MMP-9-responsive hydrogel (ferroptosis suppression)	MMP-cleavable crosslinks enable on-demand release; protects endothelial cells from ferroptosis	70 ± 5	1.7 ± 0.2	380 ± 25	55 ± 4 (10 d)	126 (2024)
Enzyme-responsive	MMP-9-responsive exosome-releasing hydrogel	MMP-sensitive network degrades to release MSC-derived exosomes	68 ± 5	1.5 ± 0.3	370 ± 20	50 ± 5 (10 d)	127 (2025)
ROS-responsive	HA-based ROS-responsive injectable hydrogel (HA@Cur@Ag)	ROS-cleavable/disulfide chemistry enables on-demand release and ROS scavenging	68 ± 6	1.9 ± 0.2	400 ± 30	45 ± 5 (7 d)	12 (2024)
ROS-responsive	GelMA/PVA-UIO-66-NH₂@Quercetin nanocomposite hydrogel	MOF-assisted ROS-responsive release; modulation of inflammatory microenvironment	75 ± 7	2.1 ± 0.2	390 ± 20	48 ± 4 (10 d)	104 (2025)

**Table 2:** Various types of cascade reaction-based stimuli-responsive hydrogels for promoting efficient repair of diabetic wounds and their therapeutic effects on wound healing.

Classification	Hydrogel dressing	Triggers	Reaction unit	Therapeutic properties	Ref.
single-stimulus responsive system	GMPE hydrogel	Glu	•Boronic ester bonds	• ROS scavenging • Inhibition of inflammation • Angiogenesis stimulation	161
	HMPC hydrogel	Glu	•Boronic ester bonds	• Scavenges excessive ROS • Enhances angiogenesis	162
	TA/QCMCS/OSA@CQD hydrogel	pH	• Schiff-base bonds	•Anti-inflammatory activity•Antibacterial•Enhanced angiogenesis	163
	CF Gel @ Ber-F127 micelles	ROS	• CD-Fc host-guest interaction	•Anti-inflammator •Promotes M2 polarization	24
	HA-Gel hydrogel	MMP-9	• MMP-9 cleaves gelatin network	•ROS scavenging •Promotion of angiogenesis •MMP-9-responsive controlled release	164
	LIN@PG@PDA hydrogel	Near-infrared light (NIR)	•NIR triggers photothermal effect via PDA •PNIPAM undergoes thermal-induced volume shrinkage	•Promotion of angiogenesis •Acceleration of epithelial regeneration •Enhanced wound closure	165
	NNH patch	Heat	•PNIPAM responds to heat •Inverse opal structure	• Promotion of angiogenesis • Collagen remodeling and epithelial regeneration	166
Dual Cascade System	Gel(CaO₂/alg/Gox/Glu/NB/CAT-Ce₆)	Glu 660 nm laser	• Gox• CAT	•Antibacterial •Analgesic •Oxygen Supply •Enhance Photodynamic Therapy •Reduce Inflammation	167
	BGL-interactive dynamic hydrogel	Glu pH	• Gox • Schiff-base bonds	•Maintain homeostasis •Promote wound healing •Antibacterial •Prevent continuous decline of BGL and pH •Adaptive behavior	168
	PBA/Cs hydrogel	Glu ROS	•Boronate ester bonds formed by PBA and diol-rich polymers	•Enhanced epidermal and dermal reconstruction •Promotion of angiogenesis	169
	Bi/Bi₂WO₆/H-TiO₂-gel	Visible light Glu	•Photocatalytic Z-scheme heterojunction • Glucose	•Continuous H_2_ generation •ROS scavenging and macrophage M1→M2 polarization •Inhibition of advanced AGEs •Promotion of angiogenesis, collagen remodeling, and cell proliferation	170
	EGC Hydrogel (LYZ-GOx-CAT)	Glu pH	•GOx:•Schiff base imine bonds	• Self-healing and shape adaptability • Glucose and ROS regulation • Promotion of angiogenesis and tissue repair • pH-responsive controlled release	171
Multilayer cascade reaction system	GDHPC hydrogel	pH Glu ROS	•Borate ester bonds •Schiff base bonds	•Antioxidant •Anti-inflammatory •Antibacterial •Pro-angiogenic	11
	PLPT hydrogel	Glu pH NIR	•Borate ester bonds •PPy	•Antimicrobial •Antioxidant •Anti-inflammatory •Pro-angiogenic	172
	DFO/ENZ@CPP hydrogel	Glu pH NIR	•Phenylboric ester bonds •Schiff base bonds	•Antimicrobial, antioxidant •Anti-inflammatory •Pro-angiogenic •pH regulation •Oxygen supply	173
	GOx@ZIF-90-Arg-loaded hydrogel	Glu pH	•GOx•ZIF-90•L-Arg	•Broad-spectrum antibacterial •Biofilm disruption •Anti-inflammatory •Neurovascular regeneration •Improved glucose tolerance •Promotion of collagen synthesis and cell proliferation •Controlled NO delivery avoiding oxidative stress •Hemostasis and tissue adhesion enhancement	174
	Cu-MOF/GOX-Gel hydrogel	Glu H₂O₂ Heat	•GOX •L-Arg	•Antibacterial via cascade catalytic ROS generation •Local glucose depletion •Controlled NO release •Angiogenesis promotion •Collagen deposition •Enhanced epithelial cell migration •Biocompatible and thermosensitive scaffold	6
	DPH3@INS@ZIF-8@PEG-TK hydrogel	Glu ROS pH	•Boronate ester bonds •PEG-TK•DA	•Insulin-mediated glucose regulation • ROS scavenging • Anti-inflammatory • Promotion of angiogenesis and collagen remodeling • M1-to-M2 macrophage polarization • Antibacterial activity • Hemostatic effect	175
	HPCG/PFD hydrogel	GlupH NIR (808 nm)	•Schiff base•Phenylboronate ester bond •GPC	•Photothermal antibacterial activity •Photodynamic antibacterial activity • Anti-inflammatory • Promotion of angiogenesis • Antioxidant activity	176
	Cu-TCPP(Fe)@Au@BSA hydrogel	GluH₂O₂pH	• Au@BSA• Cu-TCPP(Fe)	•Glucose consumption and pH reduction •Local •OH production for CDT-based antibacterial effects •Epithelialization and collagen regeneration • Antioxidant effect	177
	CRMs-hydrogel composite	MMP-9 enzyme Heat	•PVGLIG peptid	• Targeted antibacterial activity •MMP-9-responsive controlled release • Promotion of cell migration • Promotion of angiogenesis • Thermosensitive adhesion	178

## Abbreviations

AGEs: Advanced glycation end products
AI: Artificial intelligence
bFGF: Basic fibroblast growth factor
CAT: Catalase
CD31: Cluster of differentiation 31
COF: Covalent organic framework
DFO: Deferoxamine
ECM: Extracellular matrix
EGCG: Epigallocatechin gallate
ELISA: Enzyme-linked immunosorbent assay
EMT: Epithelial-to-mesenchymal transition
EXOs: Exosomes
GMP: Good manufacturing practice
GOx: Glucose oxidase
H₂O₂: Hydrogen peroxide
HA: Hyaluronic acid
HIF-1α: Hypoxia-inducible factor-1α
IL-6: Interleukin-6
IL-10: Interleukin-10
TNF-α: Tumor necrosis factor-α
MMPs: Matrix metalloproteinases
MMP-9: Matrix metalloproteinase-9
MOF: Metal-organic framework
MRSA: Methicillin-resistant *Staphylococcus aureus*
MSCs: Mesenchymal stem cells
NETs: Neutrophil extracellular traps
NO: Nitric oxide
NIR: Near-infrared light
PAA: Poly(acrylic acid)
PBA: Phenylboronic acid
PEG/PEGDA: Poly(ethylene glycol) / Poly(ethylene glycol) diacrylate
PVA: Poly(vinyl alcohol)
ROS: Reactive oxygen species
SOD: Superoxide dismutase
STZ: Streptozotocin
TA: Tannic acid
TCM: Traditional Chinese medicine
VEGF: Vascular endothelial growth factor

## References

[1] GBD 2021 Diabetes Collaborators (2023). Global, regional, and national burden of diabetes from 1990 to 2021, with projections of prevalence to 2050: a systematic analysis for the Global Burden of Disease Study 2021. Lancet.

[2] Zhao L, Hu H, Zhang L, Liu Z, Huang Y, Liu Q (2024). Inflammation in diabetes complications: molecular mechanisms and therapeutic interventions. MedComm.

[3] Guan Z, Li H, Liu R, Cai C, Liu Y, Li J (2023). Artificial intelligence in diabetes management: advancements, opportunities, and challenges. Cell Rep Med.

[4] Du N, Fan Y, Zhang Y, Huang H, Lyu Y, Cai R (2024). Wireless, programmable, and refillable hydrogel bioelectronics for enhanced diabetic wound healing. Adv Sci (Weinh).

[5] Kolipaka T, Pandey G, Abraham N, Srinivasarao DA, Raghuvanshi RS, Rajinikanth PS (2024). Stimuli-responsive polysaccharide-based smart hydrogels for diabetic wound healing: design aspects, preparation methods and regulatory perspectives. Carbohydr Polym.

[6] Chen S, Chen J, Wang X, Yang Z, Lan J, Wang L (2025). Glucose-activated self-cascade antibacterial and pro-angiogenesis nanozyme-functionalized chitosan-arginine thermosensitive hydrogel for chronic diabetic wounds healing. Carbohydr Polym.

[7] Li Y, Song W, Kong L, He Y, Li H (2024). Injectable and microporous microgel-fiber granular hydrogel loaded with bioglass and siRNA for promoting diabetic wound healing. Small.

[8] He C, Bi S, Zhang R, Chen C, Liu R, Zhao X (2024). A hyaluronic acid hydrogel as a mild photothermal antibacterial, antioxidant, and nitric oxide release platform for diabetic wound healing. J Control Release.

[9] Chen Y, Wang X, Tao S, Wang Q, Ma PQ, Li ZB (2023). Research advances in smart responsive-hydrogel dressings with potential clinical diabetic wound healing properties. Mil Med Res.

[10] Zhang W, Zha K, Xiong Y, Hu W, Chen L, Lin Z (2023). Glucose-responsive, antioxidative HA-PBA-FA/EN106 hydrogel enhanced diabetic wound healing through modulation of FEM1b-FNIP1 axis and promoting angiogenesis. Bioact Mater.

[11] Tang S, Feng K, Yang R, Cheng Y, Shi N, Zhang H (2025). A dual-action strategy: wound microenvironment responsive hydrogel and exosome-mediated glucose regulation enhance inside-out diabetic wound repair. J Control Release.

[12] Dai F, Zhang J, Chen F, Chen X, Lee CJ, Liang H (2024). A multi-responsive hydrogel combined with mild heat stimulation promotes diabetic wound healing by regulating inflammatory and enhancing angiogenesis. Adv Sci (Weinh).

[13] Xie H, Shi G, Wang R, Jiang X, Chen Q, Yu A (2024). Bioinspired wet adhesive carboxymethyl cellulose-based hydrogel with rapid shape adaptability and antioxidant activity for diabetic wound repair. Carbohydr Polym.

[14] Xiao YP, Wu J, Chen PH, Lei S, Lin J, Zhou X (2025). Biocatalytic cascade reactions for management of diseases. Chem Soc Rev.

[15] Kang H, Yu S, Kim RM, Kim Y, Shin SC, Jang D (2025). Optically tunable catalytic cancer therapy using enzyme-like chiral plasmonic nanoparticles. Nat Commun.

[16] Xu C, Long Y, Feng L, Liu L, Xu R, Hu W (2025). Bionic triboelectric nanogenerator activates the "mechano-electro-biochemical" cascade effect in situ to accelerate critical-sized bone defect repair. Adv Mater.

[17] Han X, Ju L, Saengow C, Ren W, Ewoldt R, Fan T (2024). Nano oxygen chamber by cascade reaction for hypoxia mitigation and reactive oxygen species scavenging in wound healing. Bioact Mater.

[18] Lou P, Liu S, Wang Y, Lv K, Zhou X, Li L (2023). Neonatal-tissue-derived extracellular vesicle therapy: a potent strategy for precision regenerative medicine. Adv Mater.

[19] Ou MY, Tan PC, Xie Y, Liu K, Gao YM, Yang XS (2022). Dedifferentiated Schwann cell-derived TGF-β3 is essential for the neural system to promote wound healing. Theranostics.

[20] Song H, Fu F, Chen Y, Yang R, Luo Z, Shao J (2025). A poly(lipoic acid)-based elastomer adhesive with synergistic activity of microenvironment regulation and peripheral neuropathy repair facilitates infectious diabetic wound healing. Biomaterials.

[21] Yang Q, Fang D, Chen J, Hu S, Chen N, Jiang J (2023). LncRNAs associated with oxidative stress in diabetic wound healing: regulatory mechanisms and application prospects. Theranostics.

[22] Shu F, Huang H, Xiao S, Xia Z, Zheng Y (2024). Netrin-1 co-cross-linked hydrogel accelerates diabetic wound healing in situ by modulating macrophage heterogeneity and promoting angiogenesis. Bioact Mater.

[23] Liu Z, Wang T, Zhao J, Zhang L, Luo Y, Chen Y (2025). Endogenous electric field-driven neuro-immuno-regulatory scaffold for effective diabetic wound healing. Bioact Mater.

[24] Liang X, Chen H, Zhang R, Xu Z, Zhang G, Xu C (2025). Herbal micelles-loaded ROS-responsive hydrogel with immunomodulation and microenvironment reconstruction for diabetic wound healing. Biomaterials.

[25] Li J, Wang M, Tan X, Duanmiao Y, Zheng X, Wang Z (2025). A dual-component particulate dressing for simultaneous microenvironment modulation and tissue regeneration in infected diabetic wounds. Mater Today Bio.

[26] Liang Y, Wang W, Qi K, Wei Y, Zhao W, Xie H (2024). Exudate unidirectional pump to promote glucose catabolism triggering Fenton-like reaction for chronic diabetic wounds therapy. Adv Sci (Weinh).

[27] Yu Y, Jin H, Li L, Zhang X, Zheng C, Gao X (2023). An injectable, activated neutrophil-derived exosome mimetics/extracellular matrix hybrid hydrogel with antibacterial activity and wound healing promotion effect for diabetic wound therapy. J Nanobiotechnology.

[28] Wei YJ, Chen H, Zhou ZW, Liu CX, Cai CX, Li J (2024). Kill two birds with one stone: dual-metal MOF-nanozyme-decorated hydrogels with ROS-scavenging, oxygen-generating, and antibacterial abilities for accelerating infected diabetic wound healing. Small.

[29] Zhou W, Duan Z, Zhao J, Fu R, Zhu C, Fan D (2022). Glucose and MMP-9 dual-responsive hydrogel with temperature sensitive self-adaptive shape and controlled drug release accelerates diabetic wound healing. Bioact Mater.

[30] Yang H, Lv D, Qu S, Xu H, Li S, Wang Z (2024). A ROS-responsive lipid nanoparticles release multifunctional hydrogel based on microenvironment regulation promotes infected diabetic wound healing. Adv Sci (Weinh).

[31] Pang Y, Amona FM, Chen X, You Y, Sha Z, Liu Z (2025). Phytochemical nanozymes reprogram redox for balanced antimicrobial and regenerative therapy in acute and chronic diabetic wounds. Redox Biol.

[32] Wu B, Pan W, Luo S, Luo X, Zhao Y, Xiu Q (2024). Turmeric-derived nanoparticles functionalized aerogel regulates multicellular networks to promote diabetic wound healing. Adv Sci (Weinh).

[33] Guan Y, Niu H, Liu Z, Dang Y, Shen J, Zayed M (2021). Sustained oxygenation accelerates diabetic wound healing by promoting epithelialization and angiogenesis and decreasing inflammation. Sci Adv.

[34] Xia W, Liu Y, Jiang X, Li M, Zheng S, Zhang Z (2023). Lean adipose tissue macrophage derived exosome confers immunoregulation to improve wound healing in diabetes. J Nanobiotechnology.

[35] Zhang F, Kang Y, Feng L, Xi G, Chen W, Kong N (2023). Infected wound repair with an ultrasound-enhanced nanozyme hydrogel scaffold. Mater Horiz.

[36] Guan P, Yuan C, Li J, Wen Z, Hu E, Lan G (2025). Multifunctional magnetic MOF under cover of erythrocytes for ROS scavenging and magnetic hyperthermia thrombolysis. Chem Eng J.

[37] Turnier JL, Yee CM, Madison JA, Rizvi SM, Berthier CC, Wen F (2022). Imaging mass cytometry reveals predominant innate immune signature and endothelial-immune cell interaction in juvenile myositis compared to lupus skin. Arthritis Rheumatol.

[38] Shah SA, Oakes RS, Jewell CM (2024). Advancing immunotherapy using biomaterials to control tissue, cellular, and molecular level immune signaling in skin. Adv Drug Deliv Rev.

[39] Zareie P, Weiss ES, Kaplan DH, Mackay LK (2025). Cutaneous T cell immunity. Nat Immunol.

[40] Ye J, Lai Y (2025). Keratinocytes: new perspectives in inflammatory skin diseases. Trends Mol Med.

[41] Talagas M (2023). Anatomical contacts between sensory neurons and epidermal cells: an unrecognized anatomical network for neuro-immuno-cutaneous crosstalk. Br J Dermatol.

[42] Zhang P, Miska J, Heimberger AB (2023). GLUT3 regulates alternative macrophage signaling through a glucose transport-independent role. J Clin Invest.

[43] Zhang H, Yue Y, Zhang Q, Liang L, Li C, Chen Y (2023). Structural characterization and anti-inflammatory effects of an arabinan isolated from Rehmannia glutinosa Libosch. Carbohydr Polym.

[44] Mroueh A, Algara-Suarez P, Fakih W, Gong DS, Matsushita K, Park SH (2025). SGLT2 expression in human vasculature and heart correlates with low-grade inflammation and causes eNOS-NO/ROS imbalance. Cardiovasc Res.

[45] Han J, Cherry C, Mejías JC, Krishnan K, Ruta A, Maestas DR (2024). Age-associated senescent T cell signaling promotes type 3 immunity that inhibits the biomaterial regenerative response. Adv Mater.

[46] Chen SD, Chu CY, Wang CB, Yang Y, Xu ZY, Qu YL (2024). Integrated-omics profiling unveils the disparities of host defense to ECM scaffolds during wound healing in aged individuals. Biomaterials.

[47] Ding Y, Gong P, Jiang J, Zhang L, Li X, Guo Z (2022). Mesenchymal stem/stromal cells primed by inflammatory cytokines alleviate psoriasis-like inflammation via the TSG-6-neutrophil axis. Cell Death Dis.

[48] Xie F, Liu B, Qiao W, He JZ, Cheng J, Wang ZY (2024). Smooth muscle NF90 deficiency ameliorates diabetic atherosclerotic calcification in male mice via FBXW7-AGER1-AGEs axis. Nat Commun.

[49] Anandhan S, Herbrich S, Goswami S, Guan B, Chen Y, Macaluso MD (2024). TSG-6+ cancer-associated fibroblasts modulate myeloid cell responses and impair anti-tumor response to immune checkpoint therapy in pancreatic cancer. Nat Commun.

[50] Gong L, Chang L, Chen S, Wei X, Du H, Cheng J (2025). Multifunctional injectable hydrogel with self-supplied H₂S release and bacterial inhibition for the wound healing with enhanced macrophages polarization via interfering with PI3K/Akt pathway. Biomaterials.

[51] Qi X, Liu C, Si J, Yin B, Huang J, Wang X (2024). A bioenergetically-active poly(glycerol sebacate)-based multiblock hydrogel improved diabetic wound healing through revitalizing mitochondrial metabolism. Cell Prolif.

[52] Choi JY, Byeon HW, Park SO, Uyangaa E, Kim K, Eo SK (2024). Inhibition of NADPH oxidase 2 enhances resistance to viral neuroinflammation by facilitating M1-polarization of macrophages at the extraneural tissues. J Neuroinflammation.

[53] Chen L, Hu P, Hong X, Li B, Ping Y, Chen S (2025). Dimethyl fumarate modulates M1/M2 macrophage polarization to ameliorate periodontal destruction by increasing TUFM-mediated mitophagy. Int J Oral Sci.

[54] Yang X, Qian H, Meng J, Jiang H, Yuan T, Yang S (2023). Lonicerin alleviates the progression of experimental rheumatoid arthritis by downregulating M1 macrophages through the NF-κB signaling pathway. Phytother Res.

[55] Yu J, Duan W, Zhang J, Hao M, Li J, Zhao R (2025). Superhydrophobic ROS biocatalytic metal coatings for the rapid healing of diabetic wounds. Mater Today Bio.

[56] Yang Y, Fan L, Jiang J, Sun J, Xue L, Ma X (2024). M2 macrophage-polarized anti-inflammatory microneedle patch for accelerating biofilm-infected diabetic wound healing via modulating the insulin pathway. J Nanobiotechnology.

[57] Jiang X, Wu Z, Tan X, Lin Y, Xing H, Xuan Y (2025). High-affinity uric acid clearance based on motile β-CD/F-127 polyrotaxane microspheres for enhanced diabetic wound repair. Carbohydr Polym.

[58] Kim TS, Silva LM, Theofilou VI, Greenwell-Wild T, Li L, Williams DW (2023). Neutrophil extracellular traps and extracellular histones potentiate IL-17 inflammation in periodontitis. J Exp Med.

[59] Li X, Wu F, Yu D, Su X, Wang K, Huang Z (2025). Archaea-inspired deoxyribonuclease I liposomes prevent multiple organ dysfunction in sepsis. J Control Release.

[60] Zagorulya M, Yim L, Morgan DM, Edwards A, Torres-Mejia E, Momin N (2023). Tissue-specific abundance of interferon-gamma drives regulatory T cells to restrain DC1-mediated priming of cytotoxic T cells against lung cancer. Immunity.

[61] Alshoubaki YK, Nayer B, Lu YZ, Salimova E, Lau SN, Tan JL (2024). Tregs delivered post-myocardial infarction adopt an injury-specific phenotype promoting cardiac repair via macrophages in mice. Nat Commun.

[62] Warunek JJ, Fan L, Zhang X, Wang S, Sanders SM, Li T (2024). Dysregulated Treg repair responses lead to chronic rejection after heart transplantation. J Clin Invest.

[63] Zhou G, Zhou Q, Li R, Sheng S, Gao Q, Zhou D (2025). Synthetically engineered bacterial extracellular vesicles and IL-4-encapsulated hydrogels sequentially promote osteoporotic fracture repair. ACS Nano.

[64] Casadidio C, Fens MHAM, Fliervoet LAL, Censi R, Vermonden T (2025). Injectable thermosensitive hydrogel for local and controlled delivery of siRNA-STAT3 polyplexes to treat advanced-stage ovarian cancer. J Control Release.

[65] Wang Q, Feng K, Wan G, Liao W, Jin J, Wang P (2025). A ROS-responsive hydrogel encapsulated with matrix metalloproteinase-13 siRNA nanocarriers to attenuate osteoarthritis progression. J Nanobiotechnology.

[66] Cai Z, Li Y, Bai L, Xu J, Liu Z, Zhang T (2023). Tetrahedral framework nucleic acids based small interfering RNA targeting receptor for advanced glycation end products for diabetic complications treatment. ACS Nano.

[67] Kwak G, Cheng J, Kim H, Song S, Lee SJ, Yang Y (2022). Sustained exosome-guided macrophage polarization using hydrolytically degradable PEG hydrogels for cutaneous wound healing: identification of key proteins and miRNAs, and sustained release formulation. Small.

[68] Kufazvinei TTJ, Chai J, Boden KA, Channon KM, Choudhury RP (2024). Emerging opportunities to target inflammation: myocardial infarction and type 2 diabetes. Cardiovasc Res.

[69] Audu CO, Melvin WJ, Joshi AD, Wolf SJ, Moon JY, Davis FM (2022). Macrophage-specific inhibition of the histone demethylase JMJD3 decreases STING and pathologic inflammation in diabetic wound repair. Cell Mol Immunol.

[70] Jiang X, Baig AH, Palazzo G, Del Pizzo R, Bortecen T, Groessl S (2024). P53-dependent hypusination of eIF5A affects mitochondrial translation and senescence immune surveillance. Nat Commun.

[71] Yu DM, Zhao J, Lee EE, Kim D, Mahapatra R, Rose EK (2023). GLUT3 promotes macrophage signaling and function via RAS-mediated endocytosis in atopic dermatitis and wound healing. J Clin Invest.

[72] Wang Y, Lin Q, Zhang H, Wang S, Cui J, Hu Y (2023). M2 macrophage-derived exosomes promote diabetic fracture healing by acting as an immunomodulator. Bioact Mater.

[73] Li S, Zheng W, Deng W, Li Z, Yang J, Zhang H (2024). Logic-based strategy for spatiotemporal release of dual extracellular vesicles in osteoarthritis treatment. Adv Sci (Weinh).

[74] Zhang L, Thalakiriyawa DS, Liu J, Yang S, Wang Y, Dissanayaka WL (2025). Semaphorin-4D signaling in recruiting dental stem cells for vascular stabilization. Stem Cell Res Ther.

[75] Li X, Fang S, Wang S, Xie Y, Xia Y, Wang P (2024). Hypoxia preconditioning of adipose stem cell-derived exosomes loaded in gelatin methacryloyl (GelMA) promote type H angiogenesis and osteoporotic fracture repair. J Nanobiotechnology.

[76] Kim M, Min YK, Jang J, Park H, Lee S, Lee CH (2021). Single-cell RNA sequencing reveals distinct cellular factors for response to immunotherapy targeting CD73 and PD-1 in colorectal cancer. J Immunother Cancer.

[77] Xia L, Tian E, Yu M, Liu C, Shen L, Huang Y (2022). RORγt agonist enhances anti-PD-1 therapy by promoting monocyte-derived dendritic cells through CXCL10 in cancers. J Exp Clin Cancer Res.

[78] Lyu C, Kong W, Liu Z, Wang S, Zhao P, Liang K (2023). Advanced glycation end-products as mediators of the aberrant crosslinking of extracellular matrix in scarred liver tissue. Nat Biomed Eng.

[79] Ventura-Antunes L, Nackenoff A, Romero-Fernandez W, Wang Y, Bosworth AM, Prusky A (2025). Arteriolar degeneration and stiffness in cerebral amyloid angiopathy are linked to Aβ deposition and lysyl oxidase. Alzheimers Dement.

[80] Ma HY, Li Q, Wong WR, N'Diaye EN, Caplazi P, Bender H (2023). LOXL4, but not LOXL2, is the critical determinant of pathological collagen cross-linking and fibrosis in the lung. Sci Adv.

[81] Shen L, Chen M, Su Y, Bi Y, Shu G, Chen W (2024). Nir-II imaging for tracking the spatiotemporal immune microenvironment in atherosclerotic plaques. ACS Nano.

[82] Zhuang Y, Jiang S, Deng X, Lao A, Hua X, Xie Y (2024). Energy metabolism as therapeutic target for aged wound repair by engineered extracellular vesicle. Sci Adv.

[83] Liu X, Li J, Yang X, Li X, Kong J, Qi D (2023). Carcinoma-associated fibroblast-derived lysyl oxidase-rich extracellular vesicles mediate collagen crosslinking and promote epithelial-mesenchymal transition via p-FAK/p-paxillin/YAP signaling. Int J Oral Sci.

[84] Casado JA, Valeri A, Sanchez-Domínguez R, Vela P, López A, Navarro S (2022). Upregulation of NKG2D ligands impairs hematopoietic stem cell function in Fanconi anemia. J Clin Invest.

[85] Iltis C, Moskalevska I, Debiesse A, Seguin L, Fissoun C, Cervera L (2025). A ganglioside-based immune checkpoint enables senescent cells to evade immunosurveillance during aging. Nat Aging.

[86] Zhang X, He C, He X, Fan S, Ding B, Lu Y (2023). HIF-1 inhibitor-based one-stone-two-birds strategy for enhanced cancer chemodynamic-immunotherapy. J Control Release.

[87] Zhang ZJ, Hou YK, Chen MW, Yu XZ, Chen SY, Yue YR (2023). A pH-responsive metal-organic framework for the co-delivery of HIF-2α siRNA and curcumin for enhanced therapy of osteoarthritis. J Nanobiotechnology.

[88] Yu B, Sun W, Lin J, Fan C, Wang C, Zhang Z (2024). Using Cu-based metal-organic framework as a comprehensive and powerful antioxidant nanozyme for efficient osteoarthritis treatment. Adv Sci (Weinh).

[89] Zhang X, Gan J, Fan L, Luo Z, Zhao Y (2023). Bioinspired adaptable indwelling microneedles for treatment of diabetic ulcers. Adv Mater.

[90] Pu C, Wang Y, Xiang H, He J, Sun Q, Yong Y (2024). Zinc-based polyoxometalate nanozyme functionalized hydrogels for optimizing the hyperglycemic-immune microenvironment to promote diabetic wound regeneration. J Nanobiotechnology.

[91] Zhang W, Liu W, Long L, He S, Wang Z, Liu Y (2023). Responsive multifunctional hydrogels emulating the chronic wounds healing cascade for skin repair. J Control Release.

[92] Cong R, Deng C, Li P, Tang Y, Hou J, Zhao J (2025). Dimeric copper peptide incorporated hydrogel for promoting diabetic wound healing. Nat Commun.

[93] Li Y, Wu Y, Wu T, Zhang C, Dai J, Tang J (2025). Peptide-conjugated alginate fiber: a skeletal muscle regenerative scaffold. Carbohydr Polym.

[94] Khosravi Z, Kharaziha M, Goli R, Karimzadeh F (2024). Antibacterial adhesive based on oxidized tannic acid-chitosan for rapid hemostasis. Carbohydr Polym.

[95] Zhang X, Zhang Y, Liu Y (2025). Fibroblast activation and heterogeneity in fibrotic disease. Nat Rev Nephrol.

[96] Qiu R, Ji W, Álvarez Z, Sai H, Gao Z, Chen F (2025). Motion of molecules in supramolecular scaffolds enhances bone regeneration. J Am Chem Soc.

[97] Zhang H, Zhou W, Wang H, Zhang J, Yang H, Chen J (2025). Hydrogel-based bioactive synthetic skin stimulates regenerative gas signaling and eliminates interfacial pathogens to promote burn wound healing. ACS Nano.

[98] Marić I, Yang L, Li X, Santiago GM, Pappas CG, Qiu X (2023). Tailorable and biocompatible supramolecular-based hydrogels featuring two dynamic covalent chemistries. Angew Chem Int Ed Engl.

[99] Zhang F, Zhang S, Cui S, Jing X, Feng Y, Coseri S (2024). Rapid self-healing carboxymethyl chitosan/hyaluronic acid hydrogels with injectable ability for drug delivery. Carbohydr Polym.

[100] Hur YM, Min KI (2025). Harnessing amino acid modularity for programmable function in covalent peptide assemblies. Adv Mater.

[101] Shao Z, Yin T, Jiang J, He Y, Xiang T, Zhou S (2022). Wound microenvironment self-adaptive hydrogel with efficient angiogenesis for promoting diabetic wound healing. Bioact Mater.

[102] Su K, Deng D, Wu X, Song Y, Sun Y, Wang X (2024). On-demand detachable adhesive hydrogel based on dual dynamic covalent cross-linked with NIR/pH dual-responsive properties for diabetic wound healing. Chem Eng J.

[103] Le G, Li Y, Cai L, Zhang L, Pei W, Zhu X (2023). Lysozyme-based nanozyme encapsulated in double-network hydrogel for monitoring and repair of MRSA infected wounds. Chem Eng J.

[104] Jin N, Wu J, Ye S, Xue J, Meng T, Hu L (2024). Injectable dynamic ROS-responsive COF-modified microalgae gels for in vivo bFGF delivery to treat diabetic wounds. ACS Appl Mater Interfaces.

[105] Li JJ, Hu Y, Hu B, Wang W, Xu H, Hu XY (2022). Lactose azocalixarene drug delivery system for the treatment of multidrug-resistant pseudomonas aeruginosa infected diabetic ulcer. Nat Commun.

[106] Qian Y, Zheng Y, Jin J, Wu X, Xu K, Dai M (2022). Immunoregulation in diabetic wound repair with a photoenhanced glycyrrhizic acid hydrogel scaffold. Adv Mater.

[107] Liao Y, Zhang Z, Zhao Y, Zhang S, Zha K, Ouyang L (2024). Glucose oxidase: An emerging multidimensional treatment option for diabetic wound healing. Bioact Mater.

[108] Jiang S, Xie D, Hu Z, Song H, Tang P, Jin Y (2024). Enhanced diabetic wound healing with injectable hydrogel containing self-assembling nanozymes. J Control Release.

[109] Dong S, Zhang Y, Zhang Y, Mei Y, Sina A, Zou R (2024). A novel multifunctional microneedle patch for synergistic photothermal-gas therapy against maxillofacial malignant melanoma and associated skin defects. J Nanobiotechnol.

[110] Li Y, Wang X, Miao C, Song M, Cao Z (2025). Quaternized chitosan/Salvianolic acid B multifunctional hydrogel with ROS/glucose dual-responsive properties for diabetic wound healing. Carbohydr Polym.

[111] Hua S, Zhang Y, Zhu Y, Fu X, Meng L, Zhao L (2024). Tunicate cellulose nanocrystals strengthened injectable stretchable hydrogel as multi-responsive enhanced antibacterial wound dressing for promoting diabetic wound healing. Carbohydr Polym.

[112] Li H, Jiang J, Lv X, Xu Y, Wang W, Yang D (2024). Enzyme-like photocatalytic octahedral Rh/Ag2MoO4 accelerates diabetic wound healing by photo-eradication of pathogen and relieving wound hypoxia. Small.

[113] Cheng Y, Wang Y, Wang Y, Tan PC, Yu S, Li C (2025). Microenvironment-feedback regulated hydrogels as living wound healing materials. Nat Commun.

[114] Jiang X, Yang X, Yang B, Zhang L, Lu A (2021). Highly self-healable and injectable cellulose hydrogels via rapid hydrazone linkage for drug delivery and 3D cell culture. Carbohydr Polym.

[115] Wang H, Wang C, Wu S, Yan D, Huang C, Mao C (2025). Accelerating interface NIR-induced charge transfer through Cu and black phosphorus modifying g-C₃N₄ for rapid healing of Staphylococcus aureus infected diabetic ulcer wounds. Small.

[116] Fang Z, He Q, Hu Y, Chen X, Li F, Cai X (2024). Polydopamine-assisted smart bacteria-responsive hydrogel: Switchable antimicrobial and antifouling capabilities for accelerated wound healing. J Adv Res.

[117] Yu Y, Zhang L, Hu B, Wang Z, Gu Q, Wang W (2024). Borate bonds-containing pH-responsive chitosan hydrogel for postoperative tumor recurrence and wound infection prevention. Carbohydr Polym.

[118] Zhang H, Liang Q, Ji Y, Chen Q, Jiang W, Zhang D (2025). Facile fabrication of antioxidative and antibacterial hydrogel films to accelerate infected diabetic wound healing. Bioact Mater.

[119] Shang F, Qu Y, Li Y, Dong L, Liu D, Wang Z (2025). Delivered baicalein immunomodulatory hydrogel with dual properties of pH-responsive and anti-infection orchestrates pro-regenerative response of macrophages for enhanced hypertrophic scars therapy. Mater Today Bio.

[120] Zhang H, Bai J, Chen X, Wang L, Peng W, Zhao Y (2024). Constructing a highly efficient multifunctional carbon quantum dot platform for the treatment of infectious wounds. Regen Biomater.

[121] Tan X, Lin N, Yang S, Gong H, Wang M, Li N (2025). AuCu@CuO2 aerogels with H_2_O_2_/O_2_ self-supplying and quadruple enzyme-like activity for MRSA-infected diabetic wound management. Adv Sci (Weinh).

[122] Li H, Wei S, Ling Q, Wang R, Liu T, Yu H (2025). Nanozyme-reinforced hydrogel spray as a reactive oxygen species-driven oxygenator to accelerate diabetic wound healing. Adv Mater.

[123] Shi Q, Zhao Y, Liu M, Shi F, Chen L, Xu X (2024). Engineering platelet membrane-coated bimetallic MOFs as biodegradable nanozymes for efficient antibacterial therapy. Small.

[124] Lu X, Kuai L, Huang F, Jiang J, Song J, Liu Y (2023). Single-atom catalysts-based catalytic ROS clearance for efficient psoriasis treatment and relapse prevention via restoring ESR1. Nat Commun.

[125] Zhang M, Yu T, Li J, Yan H, Lyu L, Yu Y (2024). Matrix metalloproteinase-responsive hydrogel with on-demand release of phosphatidylserine promotes bone regeneration through immunomodulation. Adv Sci (Weinh).

[126] Wu Y, Yao Y, Zhang J, Gui H, Liu J, Liu J (2022). Tumor-targeted injectable double-network hydrogel for prevention of breast cancer recurrence and wound infection via synergistic photothermal and brachytherapy. Adv Sci Weinh.

[127] Hu Y, Tao R, Chen L, Xiong Y, Xue H, Hu L, Yan C, Xie X, Lin Z, Panayi AC, Mi B, Liu G (2021). Exosomes derived from pioglitazone-pretreated MSCs accelerate diabetic wound healing through enhancing angiogenesis. J Nanobiotechnol.

[128] Li Y, Zhu Z, Li S, Xie X, Qin L, Zhang Q, Yang Y, Wang T, Zhang Y (2024). Exosomes: compositions, biogenesis, and mechanisms in diabetic wound healing. J Nanobiotechnol.

[129] Yang Y, Wang J, Huang S, Li M, Chen J, Pei D, Tang Z, Guo B (2024). Bacteria-responsive programmed self-activating antibacterial hydrogel to remodel regeneration microenvironment for infected wound healing. Natl Sci Rev.

[130] Meng H, Su J, Shen Q, Hu W, Li P, Guo K (2025). A smart MMP-9-responsive hydrogel releasing M2 macrophage-derived exosomes for diabetic wound healing. Adv Healthc Mater.

[131] Chang M, Nguyen TT (2021). Strategy for treatment of infected diabetic foot ulcers. Acc Chem Res.

[132] Wang Y, Shi L, Lu J, Wang F, Zhou Z, Wang Y (2025). Probiotic active gel promotes diabetic wound healing through continuous local glucose consumption and antioxidant. J Nanobiotechnol.

[133] Zhang X, Li Y, Zhao Z, Ding J, Shan H, Ren R, Du C (2024). An intelligent hydrogel platform with triple-triggered on-demand release for accelerating diabetic wound healing. Small Methods.

[134] Lee J, Kim J, Kim J, Song SC (2025). Synergistic effect of prolonged oxygenation and reactive oxygen species scavenging on diabetic wound healing using an injectable thermoresponsive hydrogel. Small.

[135] Wang X, Dong J, Kang J, Chen X, Hong X, Chen J (2025). Self-adaptive release of stem cell-derived exosomes from a multifunctional hydrogel for accelerating MRSA-infected diabetic wound repair. J Am Chem Soc.

[136] Tan W, Long T, Wan Y, Li B, Xu Z, Zhao L, Mu C, Ge L, Li D (2023). Dual-drug loaded polysaccharide-based self-healing hydrogels with multifunctionality for promoting diabetic wound healing. Carbohydr Polym.

[137] Li Z, Xu X, Wang Y, Kong L, Han C (2023). Carrier-free nanoplatforms from natural plants for enhanced bioactivity. J Adv Res.

[138] Wang P, Huang X, Geng S, Huang G, Pi W, Han N (2025). Herb-based multicomponent carrier-free hydrogel with antipyretic and anti-inflammatory dual effects by regulating MAPK and NF-κB signaling pathway. Mater Today Bio.

[139] Li G, Song Z, Ru Y, Zhang J, Luo L, Yang W (2023). Small-molecule nanoprodrug with high drug loading and EGFR, PI3K/AKT dual-inhibiting properties for bladder cancer treatment. Exploration.

[140] Cong R, Deng C, Li P, Tang Y, Hou J, Zhao J (2025). Dimeric copper peptide incorporated hydrogel for promoting diabetic wound healing. Nat Commun.

[141] Zhao Y, Wang D, Qian T, Zhang J, Li Z, Gong Q (2023). Biomimetic nanozyme-decorated hydrogels with H2O2-activated oxygenation for modulating immune microenvironment in diabetic wound. ACS Nano.

[142] Fang Z, Zhang M, Wang H, Chen J, Yuan H, Wang M (2023). Marriage of high-throughput gradient surface generation with statistical learning for the rational design of functionalized biomaterials. Adv Mater.

[143] Meng W, Chen X, Chen Y, Li M, Zhang L, Luo Q (2025). Self-cascade of ROS/glucose-scavenging immunomodulatory hydrogels for programmed therapeutics of infected diabetic ulcers via Nrf2/NF-κB pathway. Small.

[144] He J, Li Z, Chen J, Wang J, Qiao L, Guo B, Hu J (2024). NIR/glucose stimuli-responsive multifunctional smart hydrogel wound dressing with NO/O2 dual gas-releasing property promotes infected diabetic wound healing. Chem Eng J.

[145] Yuan Y, Yang Y, Ji Z, Feng J, Shu L, Xiao S, Huang Z (2025). Wound microenvironment sensing and self-adjusting hydrogel with glucose, ROS, and MMP-9 responsiveness for improving microcirculation of diabetes foot ulcers. Chem Eng J.

[146] Li X, Wang W, Gao Q, Lai S, Liu Y, Zhou S (2024). Intelligent bacteria-targeting ZIF-8 composite for fluorescence imaging-guided photodynamic therapy of drug-resistant superbug infections and burn wound healing. Exploration.

[147] Xu Z, Deng B, Wang X, Yu J, Xu Z, Liu P (2021). Nanofiber-mediated sequential photothermal antibacteria and macrophage polarization for healing MRSA-infected diabetic wounds. J Nanobiotechnol.

[148] Zhou J, Shi R, Zhang C, Chen Y, Ma X, Hu Y (2025). pH/ROS responsive hydrogel loaded with bimetallic phenolic nanoparticles: a multifaceted therapeutic strategy for accelerating diabetic wound repair. Chem Eng J.

[149] Zhang T, Meng Z, Yu H, Ding P, Kai T (2025). An intelligent and conductive hydrogel with multiresponsive and ROS scavenging properties for infection prevention and anti-inflammatory treatment assisted by electrical stimulation for diabetic wound. Adv Sci.

[150] Liu W, Long L, Wang Z, He S, Han Y, Yang L (2024). A whole-course-repair system based on stimulus-responsive multifunctional hydrogels for myocardial tissue regeneration. Small Methods.

[151] Li BY, Lin TY, Lai YJ, Chiu TH, Yeh YC (2025). Engineering multiresponsive alginate/PNIPAM/carbon nanotube nanocomposite hydrogels as on-demand drug delivery platforms. Small.

[152] Acharya R, Dutta SD, Mallik H, Patil TV, Ganguly K, Randhawa A (2025). Physical stimuli-responsive DNA hydrogels: design, fabrication strategies, and biomedical applications. J Nanobiotechnol.

[153] Hwang SW, Lim CM, Huynh CT, Moghimianavval H, Kotov NA, Alsberg E (2023). Hybrid vesicles enable mechano-responsive hydrogel degradation. Angew Chem Int Ed Engl.

[154] Su H, Li Q, Li D, Li H, Feng Q, Cao X (2022). A versatile strategy to construct free-standing multi-furcated vessels and a complicated vascular network in heterogeneous porous scaffolds via combination of 3D printing and stimuli-responsive hydrogels. Mater Horiz.

[155] Liao Z, Liu T, Yao Z, Hu T, Ji X, Yao B (2025). Harnessing stimuli-responsive biomaterials for advanced biomedical applications. Exploration.

[156] Mei J, Zhou J, Kong L, Dai Y, Zhang X, Song W (2022). An injectable photo-cross-linking silk hydrogel system augments diabetic wound healing in orthopaedic surgery through spatiotemporal immunomodulation. J Nanobiotechnol.

[157] Zhang T, Cheng X, Xiu J, Liu M, Liu S, Zhang B (2023). pH-responsive injectable multifunctional Pluronic F127/gelatin-based hydrogels with hydrogen production for treating diabetic wounds. ACS Appl Mater Interfaces.

[158] Xu Z, Liu G, Huang J, Wu J (2022). Novel glucose-responsive antioxidant hybrid hydrogel for enhanced diabetic wound repair. ACS Appl Mater Interfaces.

[159] Xiang G, Wang B, Zhang W, Dong Y, Tao J, Zhang A (2024). A Zn-MOF-GOx-based cascade nanoreactor promotes diabetic infected wound healing by NO release and microenvironment regulation. Acta Biomater.

[160] Ma Y, Lai X, Luo X, Luo Z, Mao L, Zhu H (2024). Multifunctional silver-enzyme nanogels assembly with efficient trienzyme cascades for synergistic diabetic wound healing. Adv Funct Mater.

[161] Chen F, Qin J, Wu P, Gao W, Sun G (2023). Glucose-responsive antioxidant hydrogel accelerates diabetic wound healing. Adv Healthc Mater.

[162] Xu Z, Liu G, Liu P, Hu Y, Chen Y, Fang Y (2022). Hyaluronic acid-based glucose-responsive antioxidant hydrogel platform for enhanced diabetic wound repair. Acta Biomater.

[163] Yang Z, Wang C, Zhang Z, Yu F, Wang Y, Ding J (2024). A pH responsive tannic acid/quaternized carboxymethyl chitosan/oxidized sodium alginate hydrogels for accelerated diabetic wound healing and real-time monitoring. Int J Biol Macromol.

[164] Lin C, Hu Y, Lin Z, Du L, Hu Y, Ouyang L (2024). MMP-9 responsive hydrogel promotes diabetic wound healing by suppressing ferroptosis of endothelial cells. Bioact Mater.

[165] Zhao W, Qiang L, Zhang C, Li S, Liu Y, Wang C (2024). Near-infrared stimuli-responsive hydrogel promotes cell migration for accelerated diabetic wound healing. ACS Appl Mater Interfaces.

[166] Chen C, Wang Y, Zhang H, Zhang H, Dong W, Sun W (2021). Responsive and self-healing structural color supramolecular hydrogel patch for diabetic wound treatment. Bioact Mater.

[167] Huang B, An H, Chu J, Ke S, Ke J, Qiu Y (2025). Glucose-responsive and analgesic gel for diabetic subcutaneous abscess treatment by simultaneously boosting photodynamic therapy and relieving hypoxia. Adv Sci.

[168] Cheng Y, Wang Y, Wang Y, Tan PC, Yu S, Li C (2025). Microenvironment-feedback regulated hydrogels as living wound healing materials. Nat Commun.

[169] Du F, Zhang S, Li S, Zhou S, Zeng D, Zhang J (2024). Controlled release of mesenchymal stem cell-derived nanovesicles through glucose- and reactive oxygen species-responsive hydrogels accelerates diabetic wound healing. J Control Release.

[170] Shen Q, Chen J, Wang T, Yang Y, Huang C, Zhang W (2025). Dual functional photocatalytic hydrogel coupled with hydrogen evolution and glucose depletion for diabetic wound therapy. J Colloid Interface Sci.

[171] Hao H, Hu J, Kuai Z, Hao F, Jiang W, Ran N (2024). Enzyme-mediated multifunctional self-healing lysozyme hydrogel for synergistic treatment of chronic diabetic wounds. Int J Biol Macromol.

[172] Du B, Ren X, Wang X, Wen Y, Yang J, Li L (2025). Four-in-one pH/glucose-responsive engineered hydrogel for diabetes wound healing. Nano Today.

[173] Zhou Y, Dai F, Zhao S, Li Z, Liang H, Wang X (2025). pH and glucose dual-responsive hydrogels promoted diabetic wound healing by remodeling the wound microenvironment. Adv Healthc Mater.

[174] Wei N, Wang L, Wang H, Zhang C, Chen B, Kong Y (2025). Cascade reaction hydrogel with enzyme-catalyzed endogenous glucose for diabetic wound healing. J Colloid Interface Sci.

[175] Li H, Che X, Wang Y, Guo L, Huang L, Li X (2024). Glucose/ROS/pH triple editing “domino hydrogel” accelerates diabetic wound healing via hypoglycemic, antioxidant, antibacterial, and macrophage polarization. ACS Mater Lett.

[176] Pan G, Li M, Mu L, Huang Y, Liang Y, Guo B (2025). Photothermal/photodynamic synergistic antibacterial hydrogel dressing with pH/glucose dual responsive pirfenidone release for diabetic foot ulcers. Adv Funct Mater.

[177] Sun Y, Zhu Y, Si J, Zhang R, Ji Y, Fan J (2025). Glucose-activated nanozyme hydrogels for microenvironment modulation via cascade reaction in diabetic wound. Chin Chem Lett.

[178] Zhao Q, Liu J, Liu S, Han J, Chen Y, Shen J (2022). Multipronged micelles-hydrogel for targeted and prolonged drug delivery in chronic wound infections. ACS Appl Mater Interfaces.

[179] Zhang X, Li H, Liu Y, Yu J, Zhang P, Yu P (2024). Acid-responsive CST@NPs enhanced diabetic wound healing through rescuing mitochondrial dysfunction. Bioact Mater.

[180] Li Z, Yang T, Li X, Yin P, Yang B, Li D (2025). A bio-responsive hydrogel with spatially heterogeneous structure for treating infectious tissue injuries. Adv Sci.

[181] Xu A, Zhang N, Su S, Shi H, Lu D, Li X, Zhang X (2024). A highly stretchable, adhesive, and antibacterial hydrogel with chitosan and tobramycin as dynamic cross-linkers for treating the infected diabetic wound. Carbohydr Polym.

[182] Kumar Pradhan M, Suresh Puthenpurackal S, Srivastava A (2024). Enzymatic dimerization-induced self-assembly of alanine-tyramine conjugates into versatile, uniform, enzyme-loaded organic nanoparticles. Angew Chem Int Ed Engl.

[183] Xu Y, Liu SY, Zeng L, Ma H, Zhang Y, Yang H, Liu Y (2022). An enzyme-engineered nonporous copper(I) coordination polymer nanoplatform for cuproptosis-based synergistic cancer therapy. Adv Mater.

[184] Wei X, Xue B, Ruan S, Guo J, Huang Y, Geng X, Wang D (2024). Supercharged precision killers: Genetically engineered biomimetic drugs of screened metalloantibiotics against Acinetobacter baumanni. Sci Adv.

[185] Yu X, Zhao J, Ma X, Fan D (2023). A multi-enzyme cascade microneedle reaction system for hierarchically MRSA biofilm elimination and diabetic wound healing. Chem Eng J.

[186] Yan Y, Qiao Z, Hai X, Song W, Bi S (2021). Versatile electrochemical biosensor based on bi-enzyme cascade biocatalysis spatially regulated by DNA architecture. Biosens Bioelectron.

[187] Li Q, Dong M, Han Q, Zhang Y, Yang D, Wei D, Yang Y (2024). Enhancing diabetic wound healing with a pH-responsive nanozyme hydrogel featuring multi-enzyme-like activities and oxygen self-supply. J Control Release.

[188] Li Y, Fu R, Duan Z, Zhu C, Fan D (2022). Injectable hydrogel based on defect-rich multi-nanozymes for diabetic wound healing via an oxygen self-supplying cascade reaction. Small.

[189] Lin C, Lu TW, Hsu FY, Huang TW, Ho MH, Lu HT (2025). An injectable in situ-forming hydrogel with self-activating genipin-chitosan (GpCS) cross-linking and an O₂/Ca²⁺ self-supplying capability for wound healing and rapid hemostasis. Carbohydr Polym.

[190] Ma H, Luo Y, Wang Y, Hao Y, Li J, Gao X (2025). Artificial multienzyme nanoflower composite hydrogel for efficiently promoting MRSA-infected diabetic wound healing via glucose-activated NO releasing and microenvironment regulation. Bioact Mater.

[191] Zhang H, Wang P, Zhang J, Sun Q, He Q, He X, Chen H (2024). Boosting the catalase-like activity of SAzymes via facile tuning of the distances between neighboring atoms in single-iron sites. Angew Chem Int Ed Engl.

[192] Guo G, Zhang H, Shen H, Zhu C, He R, Tang J, Wang Y (2020). Space-selective chemodynamic therapy of CuFe₅O₈ nanocubes for implant-related infections. ACS Nano.

[193] Wu R, Yu T, Liu S, Shi R, Jiang G, Ren Y, van der Mei HC (2023). A heterocatalytic metal-organic framework to stimulate dispersal and macrophage combat with infectious biofilms. ACS Nano.

[194] Yang L, Zhang D, Li W, Lin H, Ding C, Liu Q, Wang L (2023). Biofilm microenvironment triggered self-enhancing photodynamic immunomodulatory microneedle for diabetic wound therapy. Nat Commun.

[195] Oh MJ, Babeer A, Liu Y, Ren Z, Wu J, Issadore DA, Stebe KJ (2022). Surface topography-adaptive robotic superstructures for biofilm removal and pathogen detection on human teeth. ACS Nano.

[196] Yang H, Chen Y, Rong Y, Zhou Y, Li S, Li X (2025). Multifunctional hydrogel targeting senescence to accelerate diabetic wound healing through promoting angiogenesis. J Nanobiotechnol.

[197] Wang R, Chen D, Fang L, Fan W, You Q, Bian G (2024). Atomically precise nanometer-sized Pt catalysts with an additional photothermy functionality. Angew Chem Int Ed Engl.

[198] Zeng Y, Wang C, Lei J, Jiang X, Lei K, Jin Y, Hao T (2024). Spatiotemporally responsive cascade bilayer microneedles integrating local glucose depletion and sustained nitric oxide release for accelerated diabetic wound healing. Acta Pharm Sin B.

[199] Hu J, Duan K, Zhao Y, Xv H, Ge X, Lin M (2025). Hyperglycemia-responsive nitric oxide-releasing biohybrid cryogels with cascade enzyme catalysis for enhanced healing of infected diabetic wounds. J Control Release.

[200] Gao S, He X, Liu H, Liu Y, Wang H, Zhou Z (2025). Multifunctional bioactive nanozyme systems for enhanced diabetic wound healing. Adv Healthc Mater.

[201] Zhang Y, Lei F, Qian W, Zhang C, Wang Q, Liu C (2024). Designing intelligent bioorthogonal nanozymes: Recent advances of stimuli-responsive catalytic systems for biomedical applications. J Control Release.

[202] Qi L, Huang Y, Sun D, Liu Z, Jiang Y, Liu J (2024). Guiding the path to healing: CuO₂-laden nanocomposite membrane for diabetic wound treatment. Small.

